# Use of a Bacteriophage Lysin to Identify a Novel Target for Antimicrobial Development

**DOI:** 10.1371/journal.pone.0060754

**Published:** 2013-04-10

**Authors:** Raymond Schuch, Adam J. Pelzek, Assaf Raz, Chad W. Euler, Patricia A. Ryan, Benjamin Y. Winer, Andrew Farnsworth, Shyam S. Bhaskaran, C. Erec Stebbins, Yong Xu, Adrienne Clifford, David J. Bearss, Hariprasad Vankayalapati, Allan R. Goldberg, Vincent A. Fischetti

**Affiliations:** 1 Laboratory of Bacterial Pathogenesis and Immunology, The Rockefeller University, New York, New York, United States of America; 2 Hunter College School of Health Sciences, New York, New York, United States of America; 3 Information Science Program, Cornell Lab of Ornithology, Ithaca, New York, United States of America; 4 Laboratory of Structural Microbiology, The Rockefeller University, New York, New York, United States of America; 5 Astex Pharmaceuticals, Inc. (formerly known as SuperGen, Inc.), Dublin, California, United States of America; Cairo University, Egypt

## Abstract

We identified an essential cell wall biosynthetic enzyme in *Bacillus anthracis* and an inhibitor thereof to which the organism did not spontaneously evolve measurable resistance. This work is based on the exquisite binding specificity of bacteriophage-encoded cell wall-hydrolytic lysins, which have evolved to recognize critical receptors within the bacterial cell wall. Focusing on the *B. anthracis*-specific PlyG lysin, we first identified its unique cell wall receptor and cognate biosynthetic pathway. Within this pathway, one biosynthetic enzyme, 2-epimerase, was required for both PlyG receptor expression and bacterial growth. The 2-epimerase was used to design a small-molecule inhibitor, epimerox. Epimerox prevented growth of several Gram-positive pathogens and rescued mice challenged with lethal doses of *B. anthracis*. Importantly, resistance to epimerox was not detected (<10^−11^ frequency) in *B. anthracis* and *S. aureus*. These results describe the use of phage lysins to identify promising lead molecules with reduced resistance potential for antimicrobial development.

## Introduction

The long-term and large-scale use of antibiotics in human and veterinary medicine provides a powerful selective pressure for antibiotic-resistance to arise and eventually dominate populations of human pathogenic microorganisms [1]. Spontaneous resistance to most antibiotics appears with frequencies that could range from ≤10^−8^–10^−11^ and, through a series of successive mutations, ultimately generates clinically significant resistance which can then be mobilized in an intra- and inter-species manner by genetic elements like transposons, plasmids, integrons and genomic islands [2]. The evolution of multidrug resistance and the international dissemination of epidemic clones compound the problem, highlighting the need for new antimicrobial development strategies.

Recently, interest in bacteriophages and bacteriophage products as antimicrobial agents has been renewed in order to address the problem of evolving resistance to antibiotics [3]. In particular, the use of lysins, or bacteriophage-encoded cell wall hydrolases, has received particular attention because of a potent and often species-specific bacteriolytic activity and a notable lack of bacterial resistance to lysin activity [4], [5]. In the context of a bacteriophage lifecycle inside a bacterial host, lysins are expressed during viral replication and are ultimately used to cleave peptidoglycan, lyse the bacterium, and release progeny virions. Purified recombinant lysins, on the other hand, can also be potent lytic agents outside the viral context, driving lysis “from without” of target bacteria both in vitro and in experimentally-infected animals [4], [6], [7], [8]. Potentially therapeutic lysins generally have modular structures defined by well-conserved N-terminal peptidoglycan-cleaving domains and more divergent C-terminal cell wall binding (CBD) domains that can recognize species-specific cell wall glycopolymers (CWGs). The largely universal nature of lysin-sensitive cleavage sites in the peptidoglycan, combined with an increasing understanding of roles for CWGs in maintaining cell wall integrity, is cited to explain the absence of resistance to certain lysins [5], [9].

Peptidoglycan-linked CWGs, including teichoic acids and other secondary cell wall polysaccharides, are gaining interest as targets for antimicrobial drugs [10], [11], [12] precisely because of their importance in physiology and virulence [10], [12], [13], [14], [15]. In this study, we explore the possibility of CWGs as a target for antimicrobial development using a lysin called PlyG, encoded by the γ phage of *Bacillus anthracis*. PlyG cleaves *B. anthracis* peptidoglycan in a process proposed to first require binding to a neutral polysaccharide (NPS) composed of galactose (Gal), *N*-acetylglucosamine (GlcNAc) and *N*-acetylmannosamine (ManNAc) [8], [16]. Importantly for our work, spontaneous resistance to PlyG did not occur in either wild-type *B. anthracis* (*f* <5×10^−9^ per cell) or in chemically mutagenized cells with a 1000-fold increase in antibiotic resistance [8]. For this reason, we expected PlyG, acting in a role distinct from the treatment of anthrax, could be used to find a CWG in *B. anthracis* (and its cognate biosynthetic pathway) to serve as a target for antimicrobial development. We hypothesized that if spontaneous bacterial resistance to PlyG does not occur, then perhaps chemical inhibitors for the synthesis of its CWG receptor may be less prone to evolving resistance. Toward this end, we first identified both a CWG receptor for PlyG in *B. anthracis* and an enzyme (2-epimerase) required for CWG biosynthesis and bacterial growth. A specific inhibitor of 2-epimerase, referred to as epimerox, was designed, synthesized, and ultimately shown to be a potent inhibitor of *B. anthracis* growth in vitro and of pathogenesis in mice. No high-level resistance to epimerox activity was observed during exposure to conditions favoring the rapid evolution of antibiotic resistance, supporting the attractiveness of CWG inhibitors as antimicrobial targets.

## Results and Discussion

**Figure 1:**
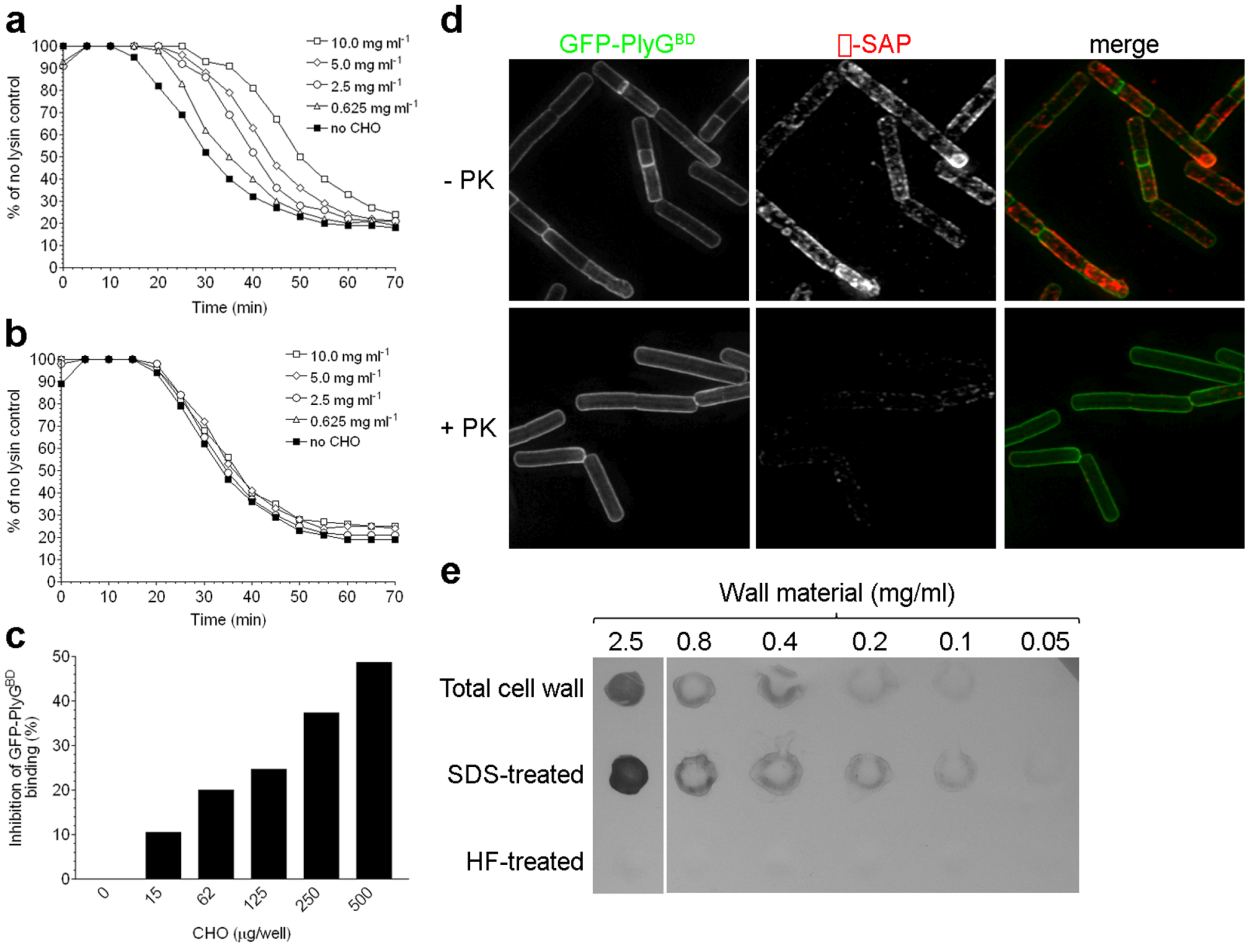
Interaction of PlyG with *B. anthracis* NPS. (A) Dose-dependent inhibition of PlyG lytic activity after pre-incubation with *B. anthracis* NPS. (B) PlyG activity after pre-incubation with increasing amounts of the CWG from *Streptococcus pyogenes*. (C) Dose-dependent inhibition of PlyG^BD^ surface-binding after pre-incubation with *B. anthracis* NPS. (D) Deltavision images of surface-labeled *B. anthracis* with or without proteinase K treatment (+/−PK). NPS (green) was labeled with GFP-PlyG^BD^, and the S-layer Sap protein (red) was labeled with specific antibodies and an Alexa Fluor 647-conjugated secondary antibody. (E) Dot-blot analysis of PlyG^BD^ binding to total cell wall material and both SDS-treated and HF-treated walls.

Our first objective was to show that PlyG binds the *B. anthracis* NPS. For this, the CWG of *B. anthracis* strain ΔSterne was purified and subjected to glycosyl composition and linkage analyses to confirm its structure. The extracted material consisted of Gal, GlcNAc, and ManNAc in the 3∶2:1 ratio (Table S1 in File S1) that defines *B. anthracis* NPS [16]. Methylation analysis also showed glycosyl linkages, including a terminally-linked Gal residue (Table S2 in File S1), consistent with *B. anthracis* [16]. We next tested whether pre-incubation of NPS with either PlyG or a GFP-labeled PlyG-binding domain (GFP-PlyG^BD^) alters subsequent lytic or cell surface-binding, respectively. Dose-dependent responses were observed in both cases, with increasing NPS levels blocking PlyG-directed lysis and binding (Figure 1A and C). Pre-incubation of PlyG with the CWG of *Streptococcus pyogenes* (a structure unrelated to *B. anthracis* NPS), however, had no effect on lytic activity (Figure 1B). As proof that PlyG likely does not bind a protein receptor, we also showed that GFP-PlyG^BD^ labels proteinase K-treated bacteria lacking most surface proteins (including the S-layer protein, Sap) (Figure 1D). Additionally, His-tagged PlyG^BD^ binds in a dose-dependent manner to purified *B. anthracis* cell wall material and SDS-treated walls (lacking most surface proteins), but not to walls extracted with hydrofluoric acid to remove CWGs (Figure 1E). Together, these findings suggest that NPS is the PlyG cell wall receptor.

**Figure 2:**
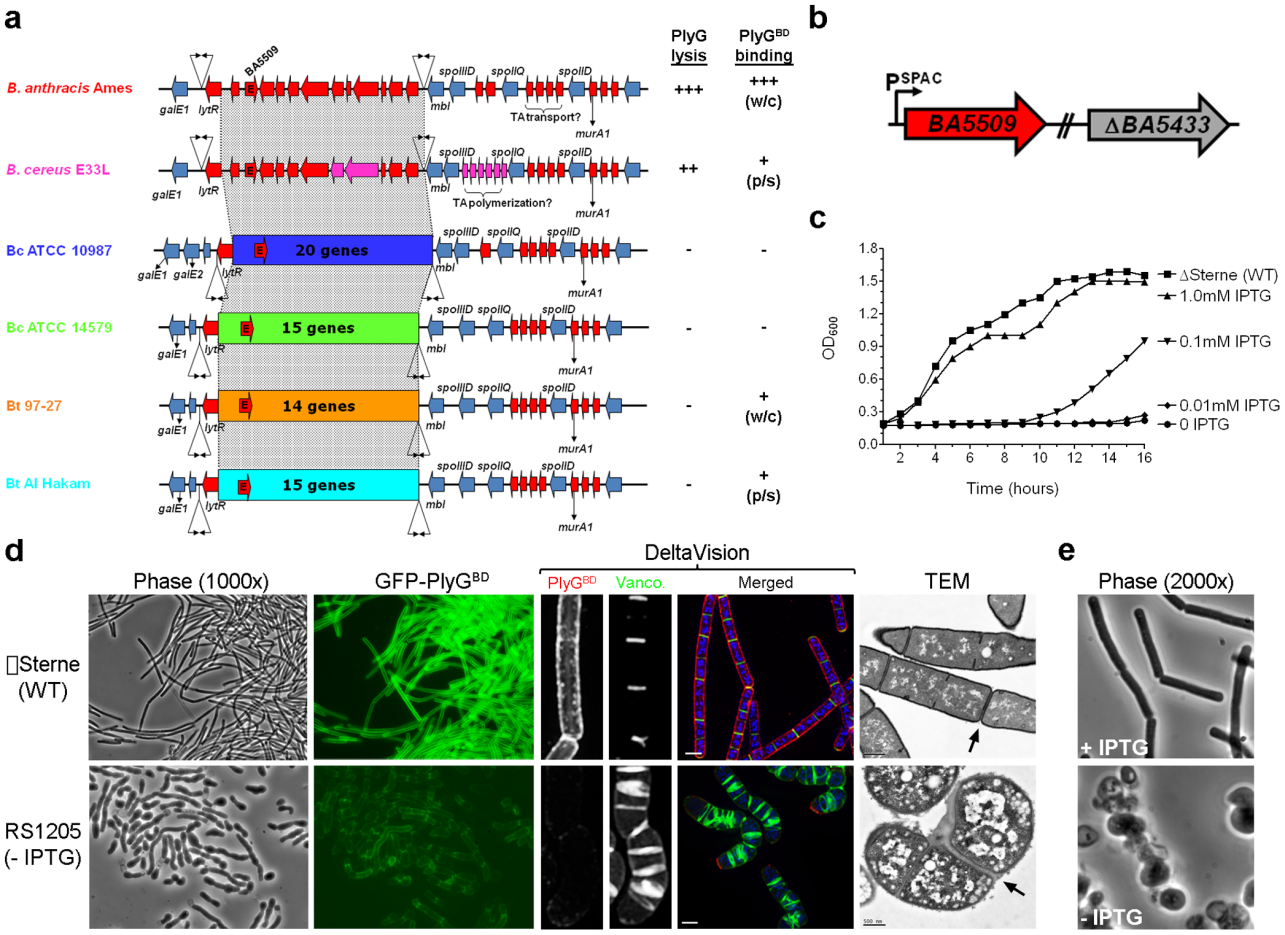
Identification and analysis of 2-epimerase in *B. anthracis*. (A) *sps* loci of the *B. cereus* lineage. Islands of variable *sps* genes are connected by gray regions and denoted by different colors. Conserved flanking sequences are shown. Red shaded loci (not in Ames) are cell wall-biosynthetic genes similar to that encoded by Ames. Inverted arrows are repeat elements. Susceptibility to PlyG lysis and GFP-PlyG^BD^ surface binding are shown. For PlyG lysis, the “+++” designation is based on an assay [8] for zones of complete bacteriolysis on agar plates overlaid with the indicated organism and treated with a 10 µl drop of PBS containing 1 µg of PlyG; “++” indicates a slight reduction in activity compared to complete bacteriolysis, and “-“ indicates an absence of bacteriolysis. For PlyG^BD^ binding, designations are based on exposure times needed to clearly visualize binding of GFP-PlyG^BD^ to target organisms by fluorescence microscopy; “+++” indicates a <5 second exposure, “+” indicates a 15–30 second exposure, and “-“ indicates no fluorescence is observed. Abbreviations: w/c, whole-cell binding; p/s, polar/septal binding. (B) Genetic representation of 2-epimerase double mutant, RS1205. (C) Growth of RS1205 (with indicated IPTG concentrations) compared to the parental wild-type strain ΔSterne. Mean averages are shown (n = 3) with standard deviations. (D) Morphological analysis of RS1205 after five hours of growth without IPTG. Phase contrast images and corresponding fluorescence fields are shown for GFP-PlyG^BD^-labeled RS1205 (5 second exposure) and *B. anthracis* ΔSterne (30 second exposure). For Deltavision images, NPS (red) was labeled with rhodamine-PlyG^BD^, division septa (green) were labeled with vancomycin BODIPY FL, and DNA (blue) was labeled with DAPI. TEM images are shown with scale bars (500 nm) and arrows denote some division septa. (E) Phase contrast microscopic images of RS1205 grown for 12 hours with and without IPTG (5 µM).

We next sought the biosynthetic pathway for *B. anthracis* NPS. A direct genomic comparison of the PlyG-sensitive *B. anthracis* Ames strain and the genetically related but PlyG-resistant strain *B. cereus* ATCC 10987 [8], [17], revealed an Ames-specific gene cluster annotated as a CWG biosynthetic pathway. Corresponding to the ∼16 kb *BA5508-BA5519* locus in *B. anthracis*, the size and gene content of this region (defined as *sps*, for surface polysaccharide synthesis) was remarkably variable over a wide range of highly related *B. cereus* group organisms (Figure 2A, Tables S3 and S4 in File S1). All *sps* loci are encoded on genetic “islands” with G+C contents distinct from their background genomes, and are flanked by nearly identical DNA sequences extending at least 5–10 kb (Tables S4 and S5 in File S1). Variation in *sps* content likely explains why CWGs with related, yet distinct glycosyl compositions are found throughout the *B. cereus* group [18]. Interestingly, *B. cereus* strain E33L, with a *sps* locus 61% identical to that of *B. anthracis*, is also sensitive to PlyG (Figure 2A). These findings support the idea that the *B. anthracis sps* locus specifies the production of NPS.

One protein, encoding a putative non-hydrolyzing UDP-*N*-acetylglucosamine 2-epimerase (or 2-epimerase), was conserved among the otherwise distinct *sps* loci in the *B*. *cereus* group (Figure 2A). The 2-epimerases are >98% identical within the *B. cereus* group and >60% identical over a range of Gram-positive organisms (Figures S1 and S2). Bacterial 2-epimerases convert UDP-GlcNAc into UDP-ManNAc prior to the polymerization of CWG subunits; epimerization is an early reaction in CWG biosynthesis and can be important or essential for growth [19], [20]. Considering the importance of 2-epimerases for bacterial viability, the broad distribution of such enzymes, and the presence of ManNAc in the *B. anthracis* lysin-inhibiting NPS, the 2-epimerse encoded by *BA5509* was chosen for further characterization.

To investigate *BA5509* as an antimicrobial target we assessed the importance of 2-epimerase to the viability of *B. anthracis*. A caveat of mutant construction, however, concerned the fact that *B. anthracis* encodes a second 2-epimerase, BA5433, which is 99% identical to BA5509 (Figure S3). The potential for functional redundancy thus required construction of a *BA5509 BA5433* double mutant (strain RS1205) (Figure 2B), in addition to single mutants. A conditional 2-epimerase mutant was first generated by placing the wild-type, monocistronic *BA5509* locus under IPTG-inducible SPAC promoter control. *BA5433* was then inactivated, in both wild-type and *BA5509* mutant backgrounds, by chromosomal integration of a recombinant plasmid. For the RS1205 double mutant, RT-PCR confirmed the IPTG-dependence for *BA5509* expression and the fact that the *BA5509* mutation did not affect expression of downstream, divergently transcribed *sps* genes (Figures S4A and S4B).

The loss of either *BA5509* or *BA5433* alone had a slight impact on *B. anthracis* growth (Fig. S5A). While the *BA5509* single mutant did have bulging cell walls and septation at inappropriate sites, GFP-PlyG^BD^ binding to surface NPS was largely unaffected (Figures S5B and S5C). The RS1205 double mutant, on the other hand, had substantial growth and morphological defects. In media supplemented with decreasing IPTG concentrations, the growth of RS1205 was arrested at 0.01 mM IPTG (Figure 2C). Microscopic examination of RS1205 revealed a progression from typical rod-shaped forms into coccoid cell-aggregates after 5 and 12 hours without IPTG (Figures 2D and 2E, Fig. S6A), in a process marked by aberrant septation and the near absence of PlyG^BD^-labeling of cell-surface NPS. Conversion into unstable coccal forms is a hallmark of mutants deficient in CWG synthesis [12], [19], [20]. These results imply that 2-epimerase is required for NPS synthesis and is important, if not essential, for *B. anthracis* viability.

**Figure 3:**
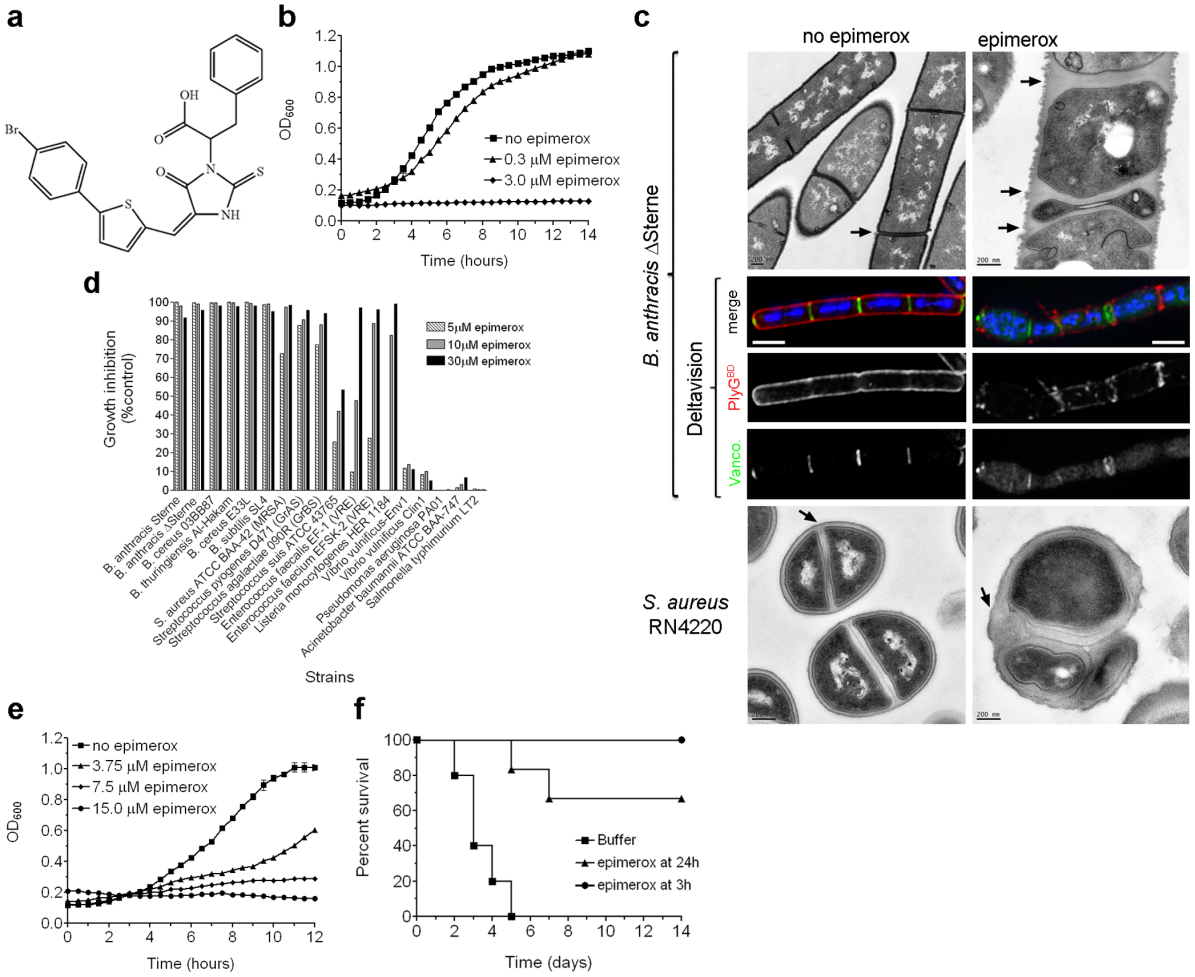
Antimicrobial activity of epimerox. (A) Chemical structure of epimerox. (B) Growth curves of *B. anthracis* ΔSterne in BHI medium with and without epimerox. (C) Morphologies of *B. anthracis* and *S. aureus* after 5 hours of exposure to epimerox (5 µM and 14 µM, respectively). For Deltavision images, NPS (red) was labeled with rhodamine-PlyG^BD^, division septa (green) were labeled with vancomycin BODIPY FL, and DNA (blue) was labeled with DAPI. For TEM images, arrows indicate some division septa. Scale bars are shown. (D) Growth inhibition assays for Gram-positive and -negative organisms. Cultures were grown in BHI medium with and without indicated epimerox concentrations for 11 hours at 28°C. (E) Growth curves of *S. aureus* strain RN4220 in BHI medium with and without epimerox. (F) Survival plot of C57BL/6 mice after i.p. infection with 5×10^5^ CFUs of *B. anthracis* Sterne, and i.p. treatment with buffer starting at 3 hours post-infection (and continuing every 6 hours for 7 days), or epimerox (13 mg/kg) starting at 3 hours or 24 hours post-infection (and continuing every 6 hours for 7 days).

In a prior publication, we described the crystal structure of BA5509 [21] and identified a novel regulatory mechanism requiring direct simultaneous interaction of the substrate molecule (UDP-GlcNAc) at both the active and allosteric sites. Having validated BA5509 as a molecule for antimicrobial development in our current study, we chose to use the allosteric site of BA5509 as the target to engineer inhibitory molecules, particularly because the allosteric site residues are conserved among bacterial 2-epimerases like BA5509 and BA5433, and, most importantly, not present in the human equivalent of the enzyme. The BA5509 allosteric site was first used in a docking model for a virtual library of ∼2,000,000 small molecules. A subset of initial compounds was identified based on calculated binding energies and predictive models for suitable drug candidates, and synthesized for testing in a *B. anthracis* growth inhibition assay. Thirty compounds, active at 30 µM, were chosen for optimization, eventually yielding 62 additional compounds for testing. The most potent inhibitor, called epimerox, is an oxo-imidizolyl compound (Figure 3A) with a minimum inhibitory concentration (MIC) of 4.0 µg ml^−1^ (7.6 µM) against both *B. anthracis* Sterne and ΔSterne strains (Table 1). Considering the well-described genetic homogeneity of all *B. anthracis* isolates [22] and the 100% identity of BA5433 and BA5509 protein sequences from over 30 distinct members of the *B. cereus* lineage of organisms, it is likely that all isolates would be susceptible to epimerox.

**Table 1:** Minimum inhibitory concentration (MIC) values for epimerox.

Organism	MIC^A^
*B. anthracis* Sterne	4.0 µg ml^−1^ (7.6 µM)
*B. anthracis* ΔSterne	4.0 µg ml^−1^ (7.6 µM)
*S. aureus* RN4220	8.0 µg ml^−1^ (16.0 µM)

A MICs were determined in triplicate on three different days using a broth.

microdilution method; no variation was observed in MIC values.

When added to *B. anthracis* cultures, epimerox effectively blocked growth for up to 14 hours at concentrations above 3 µM (Figure 3B). As with the 2-epimerase double mutant RS1205, microscopic analysis revealed a conversion from rod-shaped forms into swollen and rounded cell types after inoculation into media containing 5 µM epimerox (Figures 3C and S6B). Growth inhibition here was associated with a dramatic reduction in PlyG^BD^ surface-binding and the formation of aberrant division septa (Figure 3C). These findings are consistent with epimerox acting as a bacteriostatic agent through the inhibition of 2-epimerase activity and NPS biosynthesis in *B. anthracis* and are consistent with the phenotype seen in cells lacking both epimerase genes.

Several lines of evidence suggest epimerox acts directly upon 2-epimerase and not unintended target/s. First, in vitro docking experiments predict a very low free energy of binding (-39 kcal/mol) with the allosteric site of BA5509 and much weaker binding to the catalytic site (-16.84 kcal/mol) (Figure S7). Here, the structures of epimerox and UDP-GlcNAc are superimposable in the allosteric site and are predicted to share interactions with 15 allosteric site residues. Second, phenotypic similarities between the RS1205 epimerase double mutant (lacking 2-epimerase) and epimerox-treated cells (in which 2-epimerase is inhibited), defined by bulging filaments, aberrant septation and reduced GFP-PlyG^BD^ binding, are striking. Third, by increasing the amount of IPTG supplied to RS1205 (bearing P^SPAC^-*BA5509*) from 0.01 mM to 25 mM, the epimerox MIC shifted from 4.0 µg ml^−1^ to 8 µg ml^−1^ (Table S6 in File S1). The modest 2-fold increase in epimerox MIC observed here was associated with a 10-fold increase in 2-epimerase expression in the *BA5509 BA5433* double mutant; furthermore, the parental strain Sterne exhibited no significant change in MIC or 2-epimerase expression levels over the IPTG range. Sensitivity to epimerox was, therefore, influenced by 2-epimerase levels, over a very consistent MIC range between 4.0 µg ml^−1^ and 8 µg ml^−1^. While sensitivity levels above 8 µg ml^−1^ were not induced by 2-epimerase overexpression, 21 day serial passage resistance studies (see below) indicate 8 µg ml^−1^ is the maximum in vitro level of epimerox resistance that may be obtained. Overall, the findings support a direct interaction between epimerox and 2-epimerase. A secondary target for epimerox cannot, however, be ruled out, and would have to be both essential for viability and lacking protein sequence similarity to 2-epimerase.

While epimerox was designed against the *B. anthracis* 2-epimerase, it nonetheless inhibited the growth of many Gram-positive (but not Gram-negative) organisms that also encode 2-epimerases (Figure 3D). *Staphylococcus aureus* strain RN4220, in particular, did not grow over a 12 hour period in the presence of epimerox concentrations above 7.5 µg ml^−1^ (Figure 3E). At 7.5 µg ml^−1^ (14 µM), *S. aureus* grew very poorly and manifested cell division defects, including aberrant septal positioning and excessive cell wall material (Figure 3C), identical to that previously observed with *S. aureus* CWG mutants [15]. Since the *S. aureus* 2-epimerase differs from that of *B. anthracis* in the number of amino acids predicted to make contact with epimerox (5 of 12 contact amino acids differ in the *S. aureus* epimerase), the superior activity of epimerox against *B. anthracis* is not surprising. The activity against *S. aureus* does, however, indicate the potential in targeting bacterial 2-epimerases. Since a 2-epimerase inhibitor may not have broad-spectrum activity (as with lysins that tend to be active against single pathogenic species), other epimerox-like compounds may need to be developed to target the variations in the allosteric site of different 2-epimerases in certain Gram-positive pathogens.

Based on the potent in vitro activity of epimerox against *B. anthracis* we next tested its activity in a mouse bacteremia model after intraperitoneal (i.p.) infection. While this does not reflect the biological route of *B. anthracis* infection (or a likely route for antibiotic delivery in clinical settings), it is nonetheless a reliable and well-recognized model used in a variety of lysin efficacy studies [8], [9], [23]. In this model system, and in the absence of epimerox treatment, major organs became colonized at 3 hours post-infection with >5×10^5^ vegetative *B. anthracis* bacilli, and death occurred by 5 days (Figures 3F, S8a and S8b). When epimerox was given i.p at either 3 or 24 hours post-infection (and continuing every 6 hours for 7 days), 100% and 66% of the animals, respectively, survived 14 days. For the mice treated with epimerox at 3 hours post-infection, *B. anthracis* was detected in the major organs at 20 hours, but not after 2 days (Figure S8C). Thus, a course of epimerox therapy rescued animals infected with *B. anthracis.*

The attractiveness of epimerox as a lead molecule is enhanced by the possibility that resistance to its activity (as with PlyG) will be infrequent. Considering, however, the effect of increased BA5509 expression on the MIC of epimerox (Table S6 in File S1) and the potential influence of changes in gene dosage, we undertook a formal analysis of resistance to epimerox. First, we analyzed the appearance of spontaneous mutants resistant to epimerox and, as controls, the antibiotics rifampin and daptomycin. For both *B. anthracis* and *S. aureus*, resistance to rifampin and daptomycin was detected at frequencies between 10^−7^ and 10^−9^, respectively (Table 2). Unlike with these antibiotics however, repeated inoculations of each strain onto media supplemented with epimerox at or near MIC values, failed to yield resistant derivatives. Next, we attempted to generate epimerox-resistance using a standard serial passage method described for *S. aureus* [24]. In our study, after 6 days the epimerox MIC plateaued at 8.0 µg ml^−1^ for *B. anthracis* and 12 µg ml^−1^ for *S. aureus* from starting MICs of 4.0 µg ml^−1^ and 8.0 µg ml^−1^, respectively (Figure S9); further increases were not observed up to 21 days**.** The MIC for epimerox thus changed from 4 µg/ml to 8 µg/ml and may have been driven by an increase in *BA5509* expression akin to that observed in Table S6 of File S1 (a condition also marked by a twofold increase in epimerox MIC). High-level antibiotic resistance, similar to that observed with daptomycin by Palmer et al. [24] and here (involving an increase from 0.5 µg ml^−1^ to 18 µg ml^−1^ in only 11 days; Figure S10), was not observed with epimerox.

**Table 2:** Spontaneous antimicrobial resistance.

Treatment^A^	Organism^B^	Frequency of resistance^C^
rifampin (50 µg ml^−1^)	*B. anthracis*	3.0×10^−9^
	*S. aureus*	7.7×10^−7^
daptomycin (15 µg ml^−1^)	*B. anthracis*	1.5×10^−7^
	*S. aureus*	1.9×10^−9^
Epimerox	*B. anthracis* (3 µM)	<8.3×10^−11^
	*S. aureus* (10 µM)	<4.5×10^−11^

A Concentrated cultures were plated to agar with the indicated treatments.

B *B. anthracis* Sterne and *S. aureus* RN4220 were used in this study.

C Epimerox-resistant colonies were not observed in any experiment.

It is interesting to note that a maximum MIC for epimerox of 8 µg/mL was observed independently in both the 2-epimerase overexpression study (Table S6 in File S1) and the 21-day serial passage resistance analysis (Figure S8). The absence of resistance suggests that epimerox inhibits 2-epimerase and/or an additional target/s that is both essential for viability and encodes a 2-epimerase-like binding site. While such a target, if any, is possible, antibiotics in clinical use (i.e., quinolones and tetracyclines) can have secondary binding and inhibition targets in bacteria [25], [26], [27]. Regardless of possible secondary binding sites, the in vitro and in vivo efficacy of epimerox is striking.

Target selection is a critical consideration when developing new antimicrobial agents. It is clearly not sufficient however to choose a target based solely on its requirement for viability (i.e., the “classic” method) [28], rather targets should also be identified for which the organism has few options in circumventing, a parameter difficult to test. In this paper, we took advantage of the over billion-year co-evolution between bacteria and their phages, by exploiting the lysin-based survival strategy of one *B. anthracis*-specific phage to identify a cell wall target that may have little room to vary and evolve resistance. Indeed, the targeted and structure-based technique described here, provided us with an excellent antimicrobial compound for *B. anthracis*, epimerox, which may be further improved, with respect to potency, using a repertoire lead-optimization methodologies [29]. Our work also supports the idea that bacterial 2-epimerases in general, and perhaps even other enzymes required for the biosynthesis of lysin receptor molecules, could be viable drug targets in Gram-positive pathogens for which antibiotic resistance is a problem.

## Materials and Methods

### Ethics Statement

All in vivo protocols were approved by The Rockefeller University Institutional Animal Care and Use Committee (Protocol: 11005) and were conducted in an AAALAC accredited research facility with all efforts to minimize suffering.

### Bacterial Strains and Growth Conditions

All strains in this study, including *S. aureus* RN4220 [23] and *B. anthracis* ΔSterne and Sterne [14], [30] were grown in Brain-Heart Infusion broth (BHI; Remel). Strains with the conditional *BA5509* mutation (P^SPAC^-*BA5509*) were grown overnight with 1 mM IPTG, washed, diluted 1∶100 in BHI with or without IPTG, and grown for the indicated periods of time for analysis. Growth curves were performed in 96-well plates containing 200 µl of culture (with or without IPTG) per well; OD_600_ was recorded every 2 min (40 sec agitation between reads) for 11–20 h at 27°C in a SpectraMax Plus 96-well plate reader (Molecular Devices).

### Preparation and Analysis of Bacterial Cell Wall Carbohydrates

The isolation and purification of *B. anthracis* ΔSterne cell walls and subsequent extractions with either SDS or hydrofluoric acid (HF) were performed as described [31] with the exception that bacterial cells were initially disrupted using an EmulsiFlex C5 Homogenizer (Avestin). Glycosyl composition and linkage analyses were performed on the *B. anthracis* CWG [16]. *S. pyogenes* CWG was purified as described [32]. For the analysis of PlyG binding to purified wall material, total cell wall, SDS-extracted cell wall, and HF-extracted cell wall stocks were prepared in PBS (5 mg ml^−1^) and diluted; 70 µl aliquots of the indicated concentrations were then loaded to a dot-blot apparatus (Bio-Rad), transferred to nitrocellulose, and probed with His-tagged PlyG^BD^ (1 mg ml^−1^) [30]. After incubation with Anti-His antibody (Novagen, catalog number 70796), binding was visualized using an alkaline phosphatase-conjugated secondary antibody (Sigma, catalog number A3563).

### PlyG Inhibition Assays

PlyG (70 µl of 7.4 µg ml^−1^ stock in PBS pH 7.2) and *B. anthracis* NPS or *S. pyogenes* CWG (70 µl of indicated concentrations in PBS) were mixed for 30 min at 24°C in a 96-well plate. Lysin and/or carbohydrate were replaced with PBS alone for controls. After pre-incubation, 70 µl of log phase *B. anthracis* ΔSterne cells in PBS were added and OD_600_ was monitored every 30 sec (10 sec agitation between reads) for 70 min in a SpectraMax Plus 96-well plate reader. OD_600_ values for PlyG-treated cultures were divided by corresponding values from untreated cultures to evaluate inhibition. For inhibition of PlyG^BD^ binding, 25 µl of PlyG^BD^ (1 µg ml^−1^) and 25 µl of indicated NPS concentrations were mixed for 30 min at 24°C before addition of 75 µl of log phase ΔSterne. After 10 min, washed cell pellets were transferred to black 96-well plates to determine relative fluorescence units (RFUs) in a SpectraMax M5 plate reader (Ex = 485 nm, Em = 538 nm). RFU values for NPS-treated samples were divided by corresponding values for untreated samples to evaluate inhibition.

### *B. anthracis* Mutant Construction and Analysis

The *BA5509* promoter was replaced with the IPTG-inducible P^SPAC^ promoter as described [33]. Briefly, the first 471 bases of *BA5509* and its preceding ribosome binding site were PCR amplified with *BA5509* mutagenesis primers (Table S7 in File S1). Primer-encoded attB1 and attB2 recombinase recognition sites permitted cloning into the Gateway vector pDONRtet (Invitrogen) and transfer into pNFd13. Transformation of ΔSterne and integration into *BA5509* was performed in the presence of 5 mM IPTG. Disruption of *BA5433* was performed as described [34], using a 190 bp internal PCR fragment amplified with *BA5433* mutagenesis primers and cloned into the *Kpn*I site of plasmid pASD4. RT-PCR analysis of RS1205 was performed as described [30], using the primers in Table S7 of File S1. Quantitative PCR (qRT-PCR) analysis was performed as described [35] using primers in Table S7 of File S1 and probes for *BA5509* (5′-CCGTCGTGAAAACTT-3′) and the *rpoB* gene (5′-CTGCCGCTAAAATTT-5′); *rpoB* served as endogenous control for gene expression [36].

### Bacterial Labeling and Microscopy

Phase-contrast and fluorescence microscopy (including use of GFP-PlyG^BD^) were performed as described using an Eclipse E400 microscope (Nikon) and QCapture Pro version 5.1 software [30]. Samples for electron microscopy were stained in 0.5% uranyl acetate and viewed with a Tecnai Spirit BT Transmission Electron Microscope (FEI). The DeltaVision Image Restoration Microscope (Applied Precision) was used with non-permeabilized cells as described [37]; images are deconvolved projections of 3-dimensional data. The following stains were used: PlyG^BD^ coupled to NHS-Rhodamine (Thermo Scientific), 1 µg ml^−1^; BODIPY FL vancomycin (Invitrogen), 2.5 µg ml^-1^; DAPI, 2 µg ml^−1^; and GFP-PlyG^BD^, 1 µg ml^−1^. To digest surface proteins, overnight ΔSterne cells were treated for 2 h with chloramphenicol (10 µg ml^−1^) and proteinase K (100 µg ml^−1^), washed with PBS, and fixed in 3.75% formalin. Fixed cells were mounted on poly-L-Lysine coated slides and stained with GFP-PlyG^BD^ or anti-Sap antisera and a secondary Alexa Fluor 647-conjugated antibody (Invitrogen, catalog number A21245) as described [37].

#### Epimerox identification

Virtual screening *in silico*, in comparison with high throughput screening (HTS), can significantly decrease the number of compounds necessary for experimental assessment of activity, can increase the success rate for *in vitro* experiments, and appears to be an effective approach for finding novel hits. We employed a customized 2 million compound library having Med Chem tractability filters coupled with drug discovery software (CLIMB™) that uses entropy and electrostatic contributions along with Glide and Gold docking scoring functions [38], to target the allosteric site within the crystal structure of 2-Epimerase (PDB ID: 3BEO). A similar strategy previously identified a potent and specific inhibitor of the JAK2 protein kinase [6]. Rigid docking, free energy of molecular dynamics, structural similarity, RO5 and in vivo PK/ADME (Pharmacokinetic/Absorption, Distribution, Metabolism, Excretion) properties based custom filters used during the final phase of hit selection resulted in the output of 186 low-molecular weight molecules. One-hundred compounds from the virtual screening set were then screened in both a biochemical assay [21], and the *B. anthracis* growth inhibition assay. Numerous compounds were ultimately identified based on the ability to inhibit *B. anthracis* growth by over 50% (compared to untreated controls) at a concentration of 30 µM; the inhibitor candidates served as starting points for optimization. Based on CLIMB® guided design, 62 compounds were synthesized and tested for *B. anthracis* growth inhibition. Epimerox, the most potent inhibitor, was chosen for further pharmacological evaluation.

### Epimerox Synthesis

Epimerox, or sodium (*S*,*E*)-2-(4-((5-(3,4-dichlorophenyl)furan-2-yl)methylene)-5-oxo-2-thioxoimidazolidin-1-yl)-3-phenylpropanoate, was prepared by β-alanine catalyzed Knoevenagel condensation of substituted thiohydantoin with the corresponding aldehyde under microwave irradiation. The condensation reaction provided *E/Z* isomeric products, and the major *E* isomer was obtained by recrystallization to >99.5% purity.

### Growth Inhibition Assays

One millimolar epimerox stock solutions in 5 mM DMSO were diluted into assays at indicated concentrations. Final DMSO concentrations were always 3%. Wells of a 96-well plate contained 6 µl of inhibitor (or 6 µl of DMSO as a control), 94 µl of BHI, and 100 µl of log phase cells (OD_600_ 0.2) in BHI. OD_600_ was recorded in a SpectraMax Plus 96-well plate reader at 28°C with agitation every 2 min. Growth inhibition was calculated as follows: 100(1-[(OD_600_ at endpoint of inhibitor culture–OD_600_ of media background)/(OD_600_ at endpoint of DMSO control culture- OD_600_ of media background)]). Endpoint was defined as the entry point into stationary phase. Assays were performed in triplicate.

### Animal Model of *B. anthracis* Infection

Overnight *B. anthracis* Sterne cultures were diluted 1∶100 in BHI and grown for 3 h with aeration at 30°C. Cells were harvested, washed with sterile PBS (pH 7.2), and adjusted to a density of ∼1×10^6^ cell per ml of PBS. Infections then proceeded in a manner similar to that described [8]. Briefly, 4–6 week-old female C57BL/6 mice (fifteen per group) were infected intraperitoneally (i.p.) with 5×10^5^ bacilli. Starting at either 3 or 24 hours post- infection, epimerox was administered i.p. every six hours for up to seven days in either 20 µg (1.3 mg/kg) or 200 µg doses (13 mg/kg). Survival was monitored for 14 days. A second set of infected mice were also euthanized at indicated time points for necropsy. Heart, liver, spleen, and kidneys were excised, washed with 70% ethanol and sterile PBS (pH 7.2), homogenized in PBS, and plated on to determine viability. Uninfected mice were used to confirm the sterility of each organ. The 20 µg doses of epimerox did not rescue infected mice and results from these experiments are not shown.

### Antimicrobial Resistance Assays

MICs were determined in triplicate on three different days using the broth microdilution method as described [39]; no day-to-day variation whatsoever was observed in the MIC values reported in Table 1 and Table S6 of File S1. The MIC is the amount of drug needed to prevent growth of 5×10^5^ bacteria suspended in 0.1 ml nutrient broth and incubated in a 96-well microtiter plate at 37°C for 24 hours.

For analysis of spontaneous resistance to epimerox, rifampin (Sigma-Aldrich), and daptomycin (Tocris Bioscience), strains were grown in 100 ml BHI with agitation at 30°C (*B. anthracis* Sterne and derivatives thereof) or 37°C (*S. aureus* RN4220). After 24 hours, cultures were washed, concentrated 10-fold in media, and plated for viability on agar with or without, daptomycin (15 µg ml^−1^) rifampin (50 µg ml^−1^) or a range of epimerox concentrations. Where indicated, IPTG (at indicated concentrations) was added to both growth cultures and agar plates. Colonies appearing after 3–5 days were used to calculate resistance frequency.

Epimerox resistance was followed during serial passage in a manner similar to that described [24]. Briefly, overnight *B. anthracis* ΔSterne and *S. aureus* RN4220 cultures were adjusted to OD_600_ 0.1 in BHI medium with epimerox (ranging from 1 to 15 µM with increments of 1 µM) and grown for 18 h at 30°C with aeration. The highest epimerox concentration that yielded visible growth was washed in PBS, adjusted to OD_600_ 0.1, and aliquots were either frozen at −80°C for later analysis or incubated overnight with a range of increasing epimerox concentrations as above. While resistance values did not increase after 6 days, the experiment was ultimately continued for 21 days. After 21 days, all frozen intermediaries were revived, subcultured three times in the absence of epimerox, and the MIC of epimerox was again determined by broth microdilution. Similar experiments were performed with daptomycin and *S. aureus* strain RN4220.

## Supporting Information

Figure S1 **Protein sequence alignment of the UDP-GlcNAc 2-epimerases encoded by** ***sps*** **loci of the** ***B. cereus*** **lineage.** Alignments were obtained using ClustalW. Shading was generated by Boxshade. Black indicates 100% identical residues and gray indicates conserved amino acid changes. Proteins included are as follows: BA5509 in *B. anthracis* Ames, MnaA in *B. cereus* E33L, BCE_5307 in *B. cereus* ATCC 10987, BC5201 in *B. cereus* ATCC 14579, BT9727_4878 in *B. thuringiensis* 97–27, and BALH_4693 in *B. cereus* Al Hakam.
(DOC)

Figure S2 **Protein sequence alignment of the UDP-GlcNAc 2-epimerases encoded by different Gram-positive organisms.** Alignments were obtained using ClustalW. Shading was generated by Boxshade. Black indicates 100% identical residues and gray indicates conserved amino acid changes. Proteins included are as follows: BA5509 in *B. anthracis* strain Ames, EFWG_00415 in *Enterococcus faecium* strain Com15, MnaA (or HMPREF0348_1199) in *E. faecalis* strain TX0104, and Cap5P (or NWMN_0110) in *S. aureus* strain Newman.
(DOC)

Figure S3 **Protein sequence alignment of the BA5509 and BA5433 UDP-GlcNAc 2-epimerases encoded by** ***B. anthracis*** **.** Alignments were obtained using ClustalW. Shading was generated by Boxshade. Black indicates 100% identical or conserved residues.
(DOC)

Figure S4 **RT-PCR analysis of** ***BA5509*** **expression.** RNA was prepared after 5 hours of growth in BHI medium with and without 5 mM IPTG. The cDNA was generated and analyzed by PCR with primers specific for the indicated loci. (A) Expression of *BA5509* (and the downstream loci *BA5510* and *BA5511*) in the 2-epimerase double-mutant strain RS1205. (B) Gene expression in the wild-type *B. anthracis* strain ΔSterne. DNA size standards are shown.
(TIF)

Figure S5 **Phenotypic analysis of strains lacking the** ***BA5509*** **- or** ***BA5433*** **-encoded UDP-GlcNAc 2-epimerases of** ***B. anthracis*** **.** The *BA5509* mutant, also referred to as P^SPAC^-*BA5509*, was grown with 5 mM IPTG unless otherwise indicated. (A) Growth curve in BHI medium. (B) Phase contrast and fluorescence microscopic analysis of strains grown for 10 hours. (C) Transmission electron micrographs of strains grown for 10 hours in BHI. Scale bars are 200 nm and arrows denote some division septa.
(TIF)

Figure S6 **Ultrastructural changes associated with the inhibition or loss of UDP-GlcNAc 2-epimerase activity.** Scale bars are shown and arrows denote some division septa. (A) The *B. anthracis* ΔSterne epimerase double mutant derivative RS1205 (P^SPAC^-*BA5509*/*BA5433*::pASD4) grown for 12 hours in the absence of IPTG. (B) *B. anthracis* ΔSterne treated with epimerox (5 µM) for 5 hours at 30°C with aeration.
(TIF)

Figure S7 **A binding model (depicted with mesh representation) of epimerox using the X-ray structure of 2-epimerase (PDB entry 3BEO).** Epimerox is docked in the allosteric site with a low free energy of binding (−39 kcal/mol) and completely fills the binding site. Critical residues are labeled and Epimerox is superimosed over the natural substrate UDP-GlcNAc. Epimerox extensively overlaps the binding orientation of UDP-GlcNAc, with many putative protein contacts in common. The unsubstituted phenyl group of epimerox is positioned approximately in the location of the glucosamine of UDP-GlcNAc; here, both UDP-GlcNAc and epimerox are predicted to interact with HIS209, HIS242 and HIS44. The carboxylate and cyclic thiourea ring of epimerox overlap the positioning of UDP-GlcNAc phosphate groups, and are both predicted to interact with GLN43, GLN70, ARG211, ASN244 and GLN70. The furan ring of epimerox is roughly in the position of the ribose ring of UDP-GlcNAc (both interacting with PRO245 and ARG69), while the dichlorophenyl group of epimerox overlaps the uracil ring of UDP-Glc-NAc (both interacting with HIS44, MET47, MET243, MET66, GLN46).
(TIF)

Figure S8 **The bacterial load in epimerox treated and untreated mice.** (A) Bacteria are detected in mouse organs three hours after i.p. infection. Three mice were euthanized at 3 h and the indicated organs were removed to determine the number of colony forming units per organ. Mean values with standard deviations are shown. (B) The bacterial load in mice treated with buffer at 3 h post-infection. Samples at 20 h were taken from euthanized mice, while samples at 2–4 d were taken after death from infection. (C) Effect of epimerox treatment (administered 3 hours post infection) on the bacterial load at indicated time-points after infection with *B. anthracis*. No bacteria were detected in the organs of mice at 2 or 14 days post infection.
(TIF)

Figure S9 **Epimerox serial passage experiments.** The highest concentration of epimerox (in µg/ml) yielding growth is shown for each day of passage. No further increases were observed after six days (up to 21 days). Circles, *B. anthracis* Sterne; Squares, *S. aureus* RN4220.
(TIF)

Figure S10 **Daptomycin serial passage experiment.** The highest concentration of daptomycin (in µg/ml) yielding growth of *S. aureus* strain RN4220 is shown for each day of passage.
(TIF)

File S1 (DOCX)
