# Supplementary material for: Use of a Bacteriophage Lysin to Identify a Novel Target for Antimicrobial Development
Source: PLoS One. 2013 Apr 10;8(4):e60754. doi: 10.1371/journal.pone.0060754 (PMC3622686; doi:10.1371/journal.pone.0060754)
Supplement: Figure S1 — Protein sequence alignment of the UDP-GlcNAc 2-epimerases encoded by sps loci of the B. cereus lineage. Alignments were obtained using ClustalW. Shading was generated by Boxshade. Black indicates 100% identical residues and gray indicates conserved amino acid changes. Proteins included are as follows: BA5509 in B. anthracis Ames, MnaA in B. cereus E33L, BCE_5307 in B. cereus ATCC 10987, BC5201 in B. cereus ATCC 14579, BT9727_4878 in B. thuringiensis 97–27, and BALH_4693 in B. cereus Al Hakam. (DOC) [file pone.0060754.s001.doc]

Ba_Ames 1 MTERLKVMTIFGTRPEAIKMAPLVLELQKHPEKIESIVTVTAQHRQMLDQVLSIFGITPD
Bc_E33L 1 MTERLKVMTIFGTRPEAIKMAPLVLELQKHPEKIESIVTVTAQHRQMLDQVLSIFGITPD
Bt_97-27 1 MTERLKVMTIFGTRPEAIKMAPLVLELQKHPEKIESIVTVTAQHRQMLDQVLSIFGITPD
Bt_Al-Hakam 1 MTERLKVMTIFGTRPEAIKMAPLVLELQKHPEKIESIVTVTAQHRQMLDQVLSIFGITPD
Bc_10987 1 MTERLKVMTIFGTRPEAIKMAPLVLELQKHPEKIESIVTVTAQHRQMLDQVLSIFGITPD
Bc_14579 1 MTERLKVMTIFGTRPEAIKMAPLVLELQKHPEKIESIVTVTAQHRQMLDQVLSIFGITPD

Ba_Ames 61 FDLNIMKDRQTLIDITTRGLEGLDKVMKEAKPDIVLVHGDTTTTFIASLAAFYNQIPVGH
Bc_E33L 61 FDLNIMKDRQTLIDITTRGLEGLDKVMKEAKPDIVLVHGDTTTTFIASLAAFYNQIPVGH
Bt_97-27 61 FDLNIMKDRQTLIDITTRGLEGLDKVMKEAKPDIVLVHGDTTTTFIASLAAFYNQIPVGH
Bt_Al-Hakam 61 FDLNIMKDRQTLIDITTRGLEGLDKVMKEAKPDIVLVHGDTTTTFIASLAAFYNQIPVGH
Bc_10987 61 FDLNIMKDRQTLIDITTRGLEGLDKVMKEAKPDIVLVHGDTTTTFIASLAAFYNQIPVGH
Bc_14579 61 FDLNIMKDRQTLIDVTTRGLEGLDKVMKEAKPDIVLVHGDTTTTFIASLAAFYNQIPVGH

Ba_Ames 121 VEAGLRTWDKYSPYPEEMNRQLTGVMADLHFSPTAKSATNLQKENKDESRIFITGNTAID
Bc_E33L 121 VEAGLRTWDKYSPYPEEMNRQLTGVMADLHFSPTAKSATNLQKENKDESRIFITGNTAID
Bt_97-27 121 VEAGLRTWDKYSPYPEEMNRQLTGVMADLHFSPTAKSATNLQKENKDESRIFITGNTAID
Bt_Al-Hakam 121 VEAGLRTWDKYSPYPEEMNRQLTGVMADLHFSPTAKSATNLQKENKDESRIFITGNTAID
Bc_10987 121 VEAGLRTWDKYSPYPEEMNRQLTGVMADLHFSPTAKSATNLQKENKDESRIFITGNTAID
Bc_14579 121 VEAGLRTWDKYSPYPEEMNRQLTGVMADLHFSPTAKSATNLQKENKDESRIFITGNTAID

Ba_Ames 181 ALKTTVKETYSHPVLEKLGNNRLVLMTAHRRENLGEPMRNMFRAIKRLVDKHEDVQVVYP
Bc_E33L 181 ALKTTVKETYSHPVLEKLGNDRLVLMTAHRRENLGEPMRNMFRAIKRLVDKHEDVQVVYP
Bt_97-27 181 ALKTTVKETYSHPVLEKLGNDRLVLMTAHRRENLGEPMRNMFRAIKRLVDKHEDVQVVYP
Bt_Al-Hakam 181 ALKTTVKETYSHPVLEKLGNDRLVLMTAHRRENLGEPMRNMFRAIKRLVDKHEDVQVVYP
Bc_10987 181 ALKTTVKETYSHPVLEKLGNDRLVLMTAHRRENLGEPMRNMFRAIKRLVDKHEDVQVVYP
Bc_14579 181 ALQTTVKETYSHPVLEKLGNDRLVLMTAHRRENLGEPMRNMFRAIKRLVDKHEDVQVVYP

Ba_Ames 241 VHMNPVVRETANDILGDYGRIHLIEPLDVIDFHNVAARSYLMLTDSGGVQEEAPSLGVPV
Bc_E33L 241 VHMNPVVRETANDILGDHGRIHLIEPLDVIDFHNVAARSYLMLTDSGGVQEEAPSLGVPV
Bt_97-27 241 VHMNPVVRETANDILGDHGRIHLIEPLDVIDFHNVAARSYLMLTDSGGVQEEAPSLGVPV
Bt_Al-Hakam 241 VHMNPVVRETANDILGDHGRIHLIEPLDVIDFHNVAARSYLMLTDSGGVQEEAPSLGVPV
Bc_10987 241 VHMNPVVRETANDILGDHGRIHLIEPLDVIDFHNVAARSYLMLTDSGGVQEEAPSLGVPV
Bc_14579 241 VHMNPVVREIANEILGEHNRIHLIEPLDVIDFHNVAARSYLMLTDSGGVQEEAPSLGVPV

Ba_Ames 301 LVLRDTTERPEGIEAGTLKLAGTDEETIFSLADELLSDKEAHDKMSKASNPYGDGRASER
Bc_E33L 301 LVLRDTTERPEGIEAGTLKLAGTDEETIFSLADELLSDKKAHDKMSKASNPYGDGRASER
Bt_97-27 301 LVLRDTTERPEGIEAGTLKLAGTDEETIFSLADELLSDKEAHDKMSKASNPYGDGRASER
Bt_Al-Hakam 301 LVLRDTTERPEGIEAGTLKLAGTDEETIFSLADELLSDKEAHDKMSKASNPYGDGRASER
Bc_10987 301 LVLRDTTERPEGIEAGTLKLAGTDEETIFSLADELLSDKEAHDKMSKASNPYGDGRASER
Bc_14579 301 LVLRDTTERPEGIEAGTLKLAGTDEETIFGLADELLSDKEAHDKMAKASNPYGDGRASER

Ba_Ames 361 IVEAILKHFNK
Bc_E33L 361 IVEAILKHFNK
Bt_97-27 361 IVEAILKHFNK
Bt_Al-Hakam 361 IVEAILKHFNK
Bc_10987 361 IVEAILKHFNK
Bc_14579 361 IVEAILQHFNK
