# Supplementary material for: Use of a Bacteriophage Lysin to Identify a Novel Target for Antimicrobial Development
Source: PLoS One. 2013 Apr 10;8(4):e60754. doi: 10.1371/journal.pone.0060754 (PMC3622686; doi:10.1371/journal.pone.0060754)
Supplement: Figure S2 — Protein sequence alignment of the UDP-GlcNAc 2-epimerases encoded by different Gram-positive organisms. Alignments were obtained using ClustalW. Shading was generated by Boxshade. Black indicates 100% identical residues and gray indicates conserved amino acid changes. Proteins included are as follows: BA5509 in B. anthracis strain Ames, EFWG_00415 in Enterococcus faecium strain Com15, MnaA (or HMPREF0348_1199) in E. faecalis strain TX0104, and Cap5P (or NWMN_0110) in S. aureus strain Newman. (DOC) [file pone.0060754.s002.doc]

EFWG_00415_Efcm 1 ---------MKIKIMTIFGTRPEAIKMAPLIKAIEND-ERFESIVTVTAQHRQMLDQVMD
MnaA_Efcs 1 -------MMKKIKVMTVFGTRPEAIKMAPLIKVLEEQSEGFDSVVVVTAQHRQMLDQVLE
BA5509_Ba 1 -------MTERLKVMTIFGTRPEAIKMAPLVLELQKHPEKIESIVTVTAQHRQMLDQVLS
Cap5P_SaNewman 1 MCLNFREDNVMKKIMVIFGTRPEAIKMAPLVKEIDHN-GNFEANIVITAQHRDMLDSVLS

EFWG_00415_Efcm 51 IFDLKADYDLNIMKDGQTLTDVTSRVIKELDSVLVEAKPDIILVHGDTTTTFAASIAGFY
MnaA_Efcs 54 DFQITPNHDLNIMKDGQTLTDITSRVLNLLTEVLVTEQPDIVLVHGDTTTSFAAALAAFY
BA5509_Ba 54 IFGITPDFDLNIMKDRQTLIDITTRGLEGLDKVMKEAKPDIVLVHGDTTTTFIASLAAFY
Cap5P_SaNewman 60 IFDIQADHDLNIMQDQQTLAGLTANALAKLDSIINEEQPDMILVHGDTTTTFVGSLAAFY

EFWG_00415_Efcm 111 HQIKIGHVEAGLRTWNKYSPFPEEMNRQLTDTLADIYFAPTVMSKSNLLKEGRSEKSIFI
MnaA_Efcs 114 QQIPVGHVEAGLRTWQKYSPFPEEMNRQLVDVLTDIYFAPTTQSKGNLIKENHPEEHIYV
BA5509_Ba 114 NQIPVGHVEAGLRTWDKYSPYPEEMNRQLTGVMADLHFSPTAKSATNLQKENKDESRIFI
Cap5P_SaNewman 120 HQIPVGHVEAGLRTHQKYSPFPEELNRVMVSNIAELNFAPTVIAAKNLLFENKDKERIFI

EFWG_00415_Efcm 171 TGNTAIDAMKYTIKQNYSNDLLDNLAG-KRIILVTMHRRENLGQPMTNVFKAINRLIEKF
MnaA_Efcs 174 TGNTAIDAMAYTVDAHYQNDLLEKIPTDQRIVLITMHRRENLGLPMANVFKAVRRLVMEH
BA5509_Ba 174 TGNTAIDALKTTVKETYSHPVLEKLGN-NRLVLMTAHRRENLGEPMRNMFRAIKRLVDKH
Cap5P_SaNewman 180 TGNTVIDALSTTVQNDFVSTIINKHKG-KKVVLLTAHRRENIGEPMHQIFKAVRDLADEY

EFWG_00415_Efcm 230 EDVHIVFPMHKNPKVRKNAEETFNDSEQVHLIEPLDVIDFQNFSNNSYMILSDSGGVQEE
MnaA_Efcs 234 PEIEVIFPMHKNPKVREIVAEHLGELARVHLIEPLDVKDFQNFAAKSSLILTDSGGVQEE
BA5509_Ba 233 EDVQVVYPVHMNPVVRETANDILGDYGRIHLIEPLDVIDFHNVAARSYLMLTDSGGVQEE
Cap5P_SaNewman 239 KDVVFIYPMHRNPKVRAIAEKYLSGRNRIELIEPLDAIEFHNFTNQSYLVLTDSGGIQEE

EFWG_00415_Efcm 290 APSLGVPVLVLRDTTERPEGIEVGTLKLVGTEEDKVFEEATLLLSDKEEYKKMSQASNPY
MnaA_Efcs 294 APSLGVPVLVLRDTTERPEGVAAGTLKLVGTDEQVVYQEAKTLLTDSDAYHAMAHAQNPY
BA5509_Ba 293 APSLGVPVLVLRDTTERPEGIEAGTLKLAGTDEETIFSLADELLSDKEAHDKMSKASNPY
Cap5P_SaNewman 299 APTFGKPVLVLRNHTERPEGVEAGTSRVIGTDYDNIVRNVKQLIEDDEAYQRMSQANNPY

EFWG_00415_Efcm 350 GDGNASERILDAIAYNFGIQQEKPIDFQ-----
MnaA_Efcs 354 GDGQASHRIVEAIAYEMQQSDKKPDTFTAK---
BA5509_Ba 353 GDGRASERIVEAILKHFNK--------------
Cap5P_SaNewman 359 GDGQASRRICEAIEYYFGLRTDKPDEFVPLRHK
