# Supplementary material for: Use of a Bacteriophage Lysin to Identify a Novel Target for Antimicrobial Development
Source: PLoS One. 2013 Apr 10;8(4):e60754. doi: 10.1371/journal.pone.0060754 (PMC3622686; doi:10.1371/journal.pone.0060754)
Supplement: Figure S3 — Protein sequence alignment of the BA5509 and BA5433 UDP-GlcNAc 2-epimerases encoded by B. anthracis . Alignments were obtained using ClustalW. Shading was generated by Boxshade. Black indicates 100% identical or conserved residues. (DOC) [file pone.0060754.s003.doc]

BA5509 1 FDLNIMKDRQTLIDITTRGLEGLDKVMKEAKPDIVLVHGDTTTTFIASLAAFYNQIPVGH
BA5433 1 FDLNIMKDRQTLIDITTRGLEGLDKVMKEAKPDIVLVHGDTTTTFIASLAAFYNQIPVGH

BA5509 61 VEAGLRTWDKYSPYPEEMNRQLTGVMADLHFSPTAKSATNLQKENKDESRIFITGNTAID
BA5433 61 VEAGLRTWDKYSPYPEEMNRQLTGVMADLHFSPTAKSATNLQKENKDESRIFITGNTAID

BA5509 121 ALKTTVKETYSHPVLEKLGNNRLVLMTAHRRENLGEPMRNMFRAIKRLVDKHEDVQVVYP
BA5433 121 ALKTTVKETYSHPVLEKLGNDRLVLMTAHRRENLGEPMRNMFRAIKRLVDKHEDVQVVYP

BA5509 181 VHMNPVVRETANDILGDYGRIHLIEPLDVIDFHNVAARSYLMLTDSGGVQEEAPSLGVPV
BA5433 181 VHMNPVVRETANDILGDHGRIHLIEPLDVIDFHNVAARSYLMLTDSGGVQEEAPSLGVPA

BA5509 241 LVLRDTTERPEGIEAGTLKLAGTDEETIFSLADELLSDKEAHDKMSKASNPYGDGRASER
BA5433 241 LVLRDTTERPEGIEAGTLKLAGTDEETIFSLADELLSDKEAHDKMSKASNPYGDGRASER

BA5509 301 IVEAILKHFNK
BA5433 301 IVEAILKHFNK
