# Supplementary figures and images for: Use of a Bacteriophage Lysin to Identify a Novel Target for Antimicrobial Development
Source: PLoS One. 2013 Apr 10;8(4):e60754. doi: 10.1371/journal.pone.0060754 (PMC3622686; doi:10.1371/journal.pone.0060754)

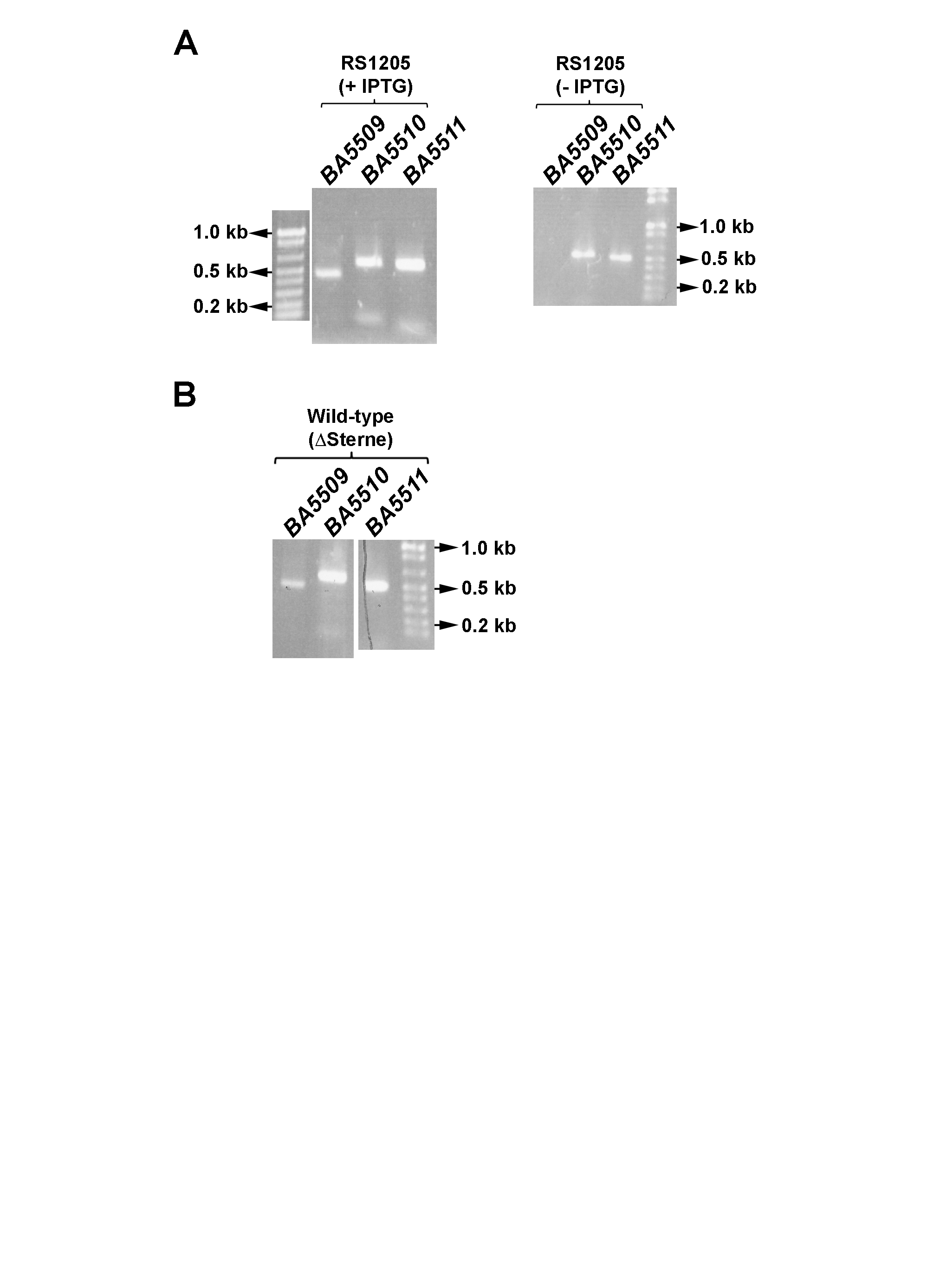

Supplement: Figure S4 — RT-PCR analysis of BA5509 expression. RNA was prepared after 5 hours of growth in BHI medium with and without 5 mM IPTG. The cDNA was generated and analyzed by PCR with primers specific for the indicated loci. (A) Expression of BA5509 (and the downstream loci BA5510 and BA5511) in the 2-epimerase double-mutant strain RS1205. (B) Gene expression in the wild-type B. anthracis strain ΔSterne. DNA size standards are shown. (TIF) [file pone.0060754.s004.tif]

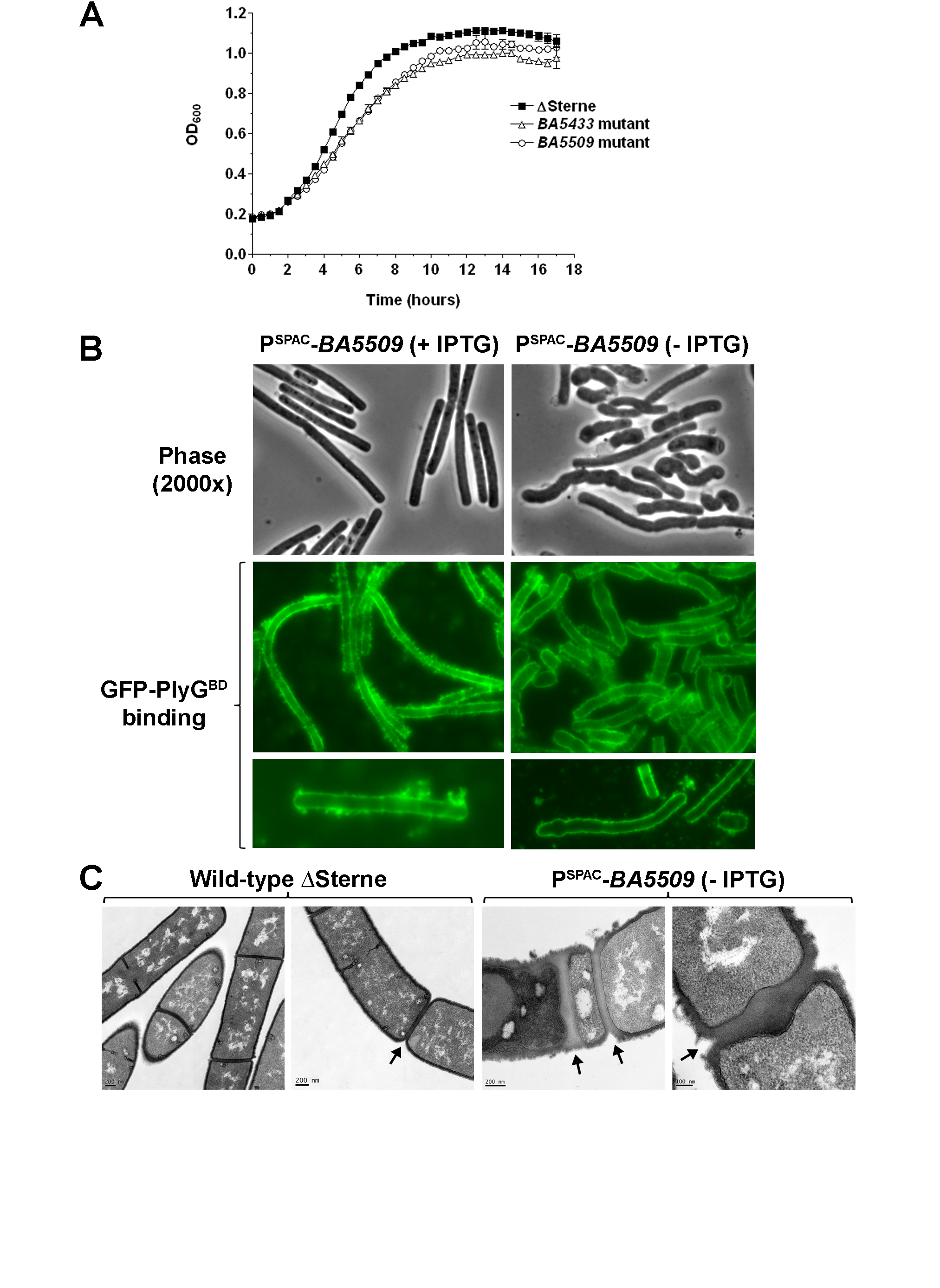

Supplement: Figure S5 — Phenotypic analysis of strains lacking the BA5509 - or BA5433 -encoded UDP-GlcNAc 2-epimerases of B. anthracis . The BA5509 mutant, also referred to as PSPAC-BA5509, was grown with 5 mM IPTG unless otherwise indicated. (A) Growth curve in BHI medium. (B) Phase contrast and fluorescence microscopic analysis of strains grown for 10 hours. (C) Transmission electron micrographs of strains grown for 10 hours in BHI. Scale bars are 200 nm and arrows denote some division septa. (TIF) [file pone.0060754.s005.tif]

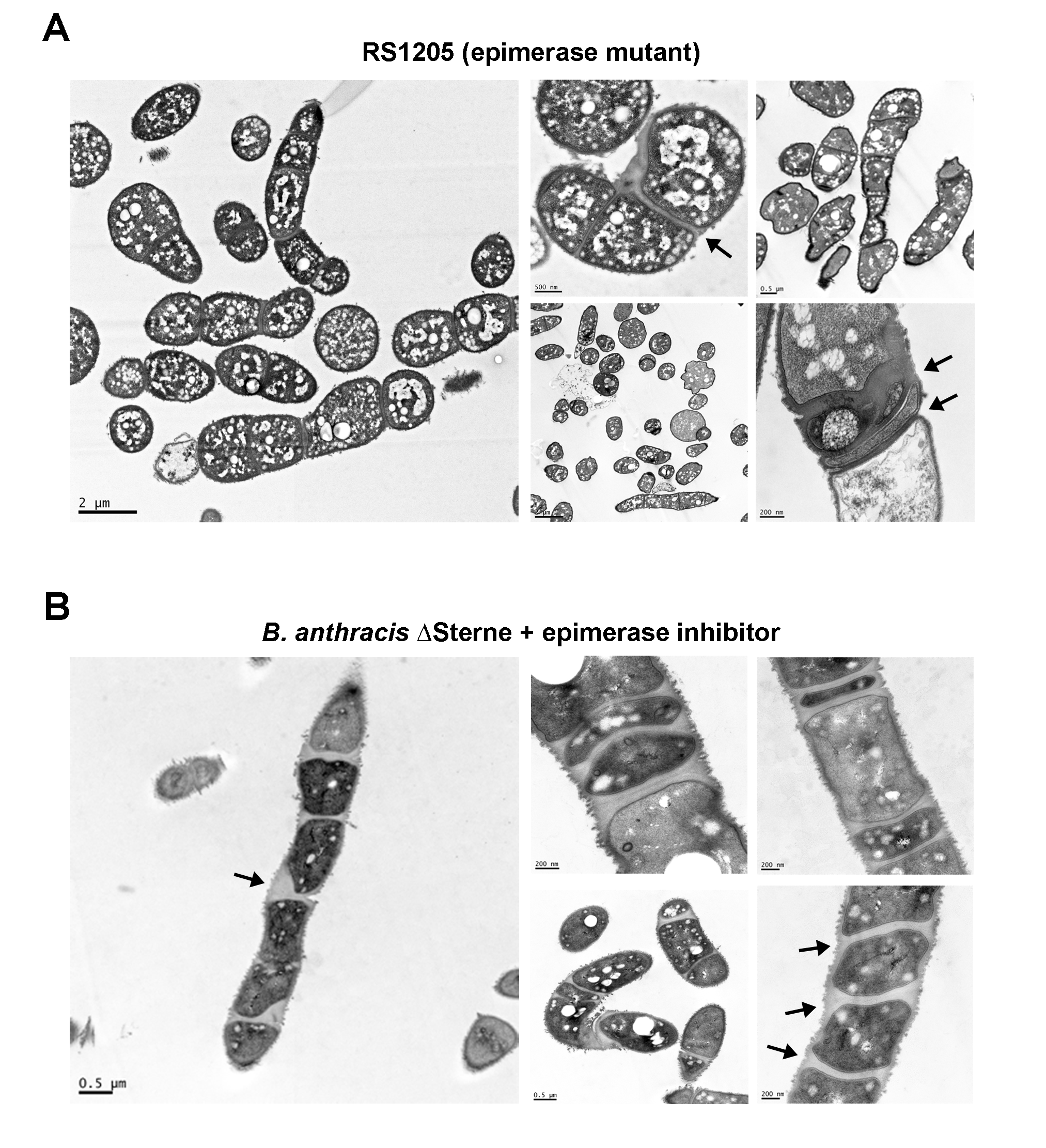

Supplement: Figure S6 — Ultrastructural changes associated with the inhibition or loss of UDP-GlcNAc 2-epimerase activity. Scale bars are shown and arrows denote some division septa. (A) The B. anthracis ΔSterne epimerase double mutant derivative RS1205 (PSPAC-BA5509/BA5433::pASD4) grown for 12 hours in the absence of IPTG. (B) B. anthracis ΔSterne treated with epimerox (5 µM) for 5 hours at 30°C with aeration. (TIF) [file pone.0060754.s006.tif]

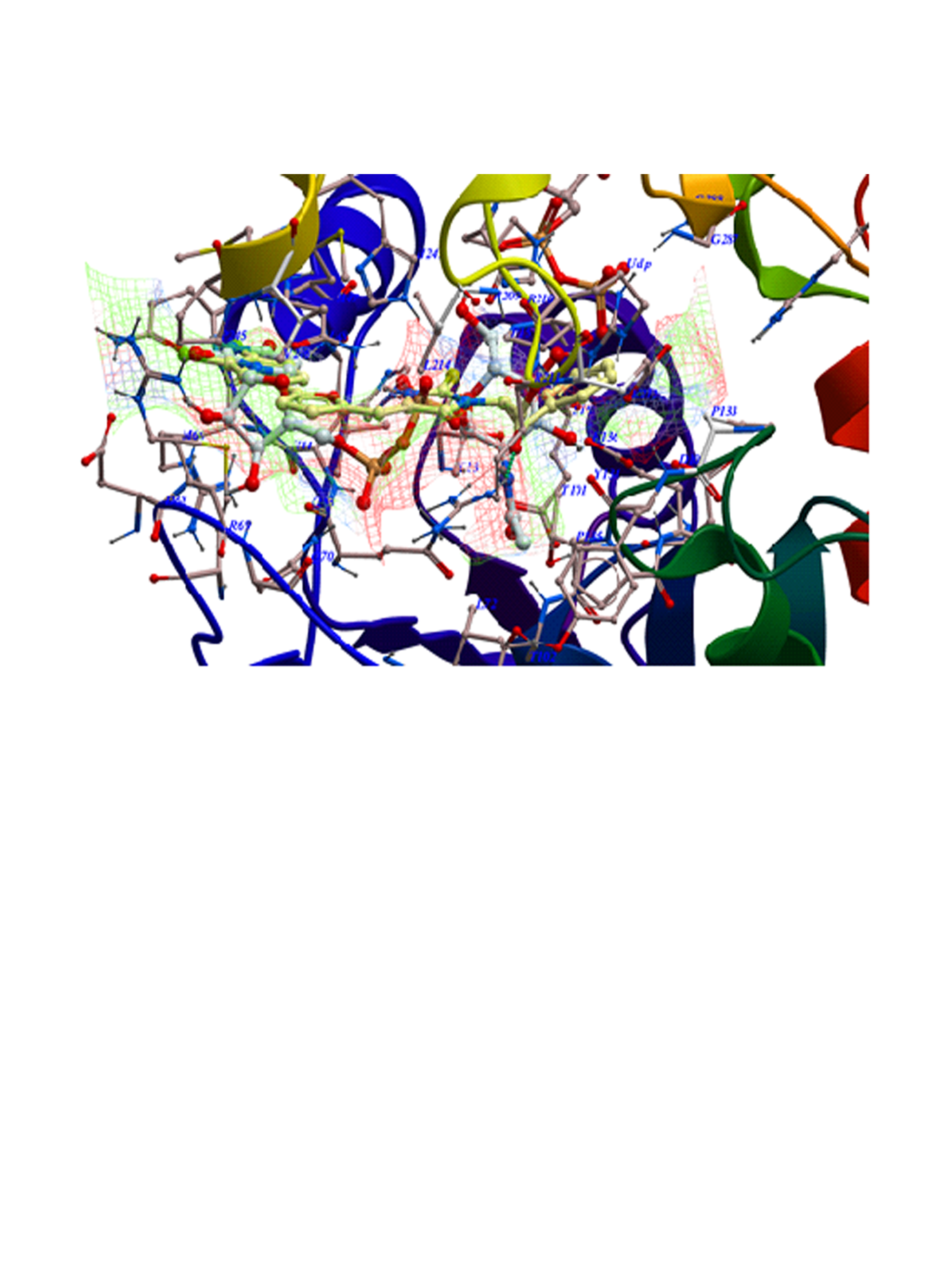

Supplement: Figure S7 — A binding model (depicted with mesh representation) of epimerox using the X-ray structure of 2-epimerase (PDB entry 3BEO). Epimerox is docked in the allosteric site with a low free energy of binding (−39 kcal/mol) and completely fills the binding site. Critical residues are labeled and Epimerox is superimosed over the natural substrate UDP-GlcNAc. Epimerox extensively overlaps the binding orientation of UDP-GlcNAc, with many putative protein contacts in common. The unsubstituted phenyl group of epimerox is positioned approximately in the location of the glucosamine of UDP-GlcNAc; here, both UDP-GlcNAc and epimerox are predicted to interact with HIS209, HIS242 and HIS44. The carboxylate and cyclic thiourea ring of epimerox overlap the positioning of UDP-GlcNAc phosphate groups, and are both predicted to interact with GLN43, GLN70, ARG211, ASN244 and GLN70. The furan ring of epimerox is roughly in the position of the ribose ring of UDP-GlcNAc (both interacting with PRO245 and ARG69), while the dichlorophenyl group of epimerox overlaps the uracil ring of UDP-Glc-NAc (both interacting with HIS44, MET47, MET243, MET66, GLN46). (TIF) [file pone.0060754.s007.tif]

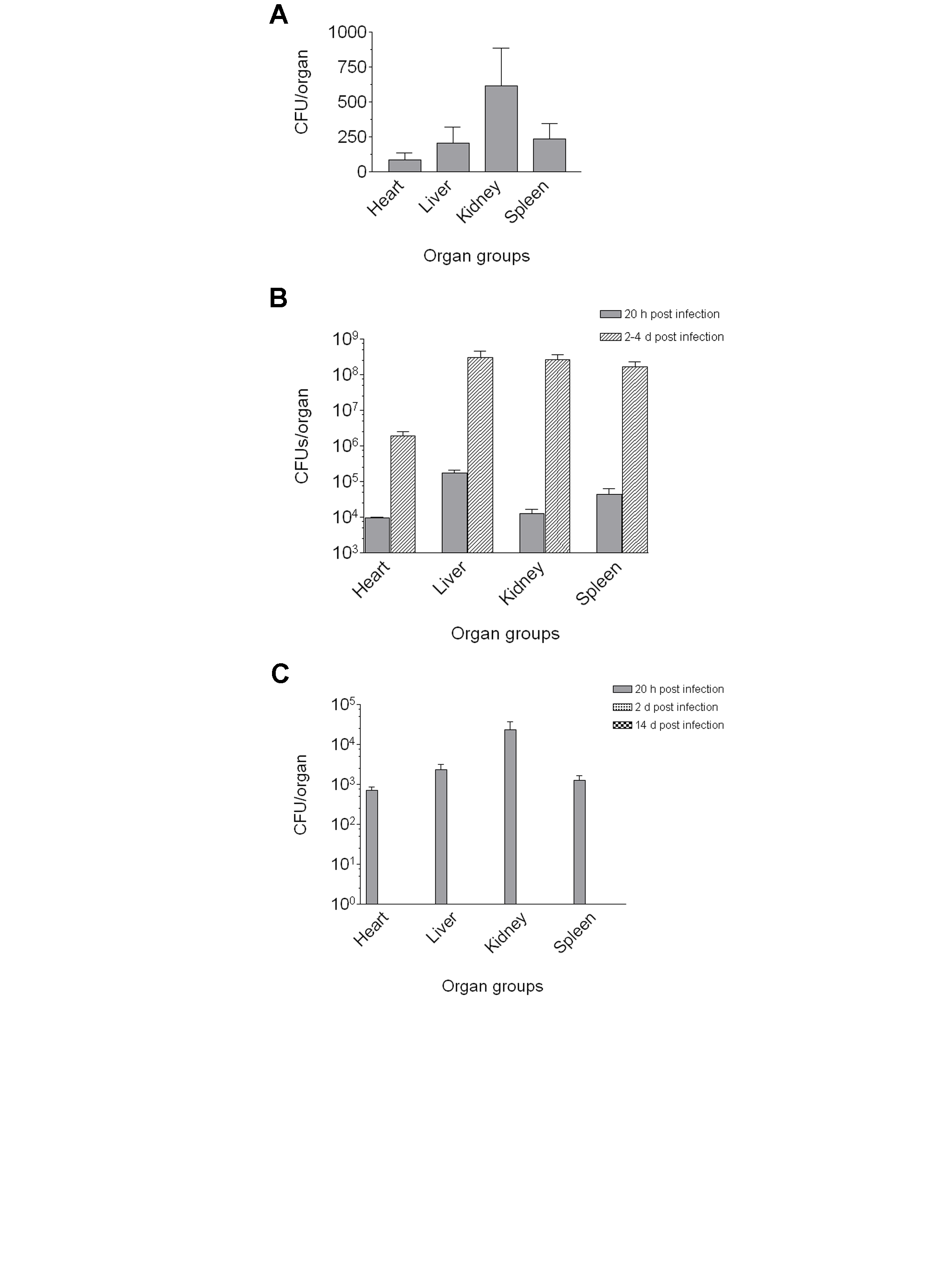

Supplement: Figure S8 — The bacterial load in epimerox treated and untreated mice. (A) Bacteria are detected in mouse organs three hours after i.p. infection. Three mice were euthanized at 3 h and the indicated organs were removed to determine the number of colony forming units per organ. Mean values with standard deviations are shown. (B) The bacterial load in mice treated with buffer at 3 h post-infection. Samples at 20 h were taken from euthanized mice, while samples at 2–4 d were taken after death from infection. (C) Effect of epimerox treatment (administered 3 hours post infection) on the bacterial load at indicated time-points after infection with B. anthracis. No bacteria were detected in the organs of mice at 2 or 14 days post infection. (TIF) [file pone.0060754.s008.tif]

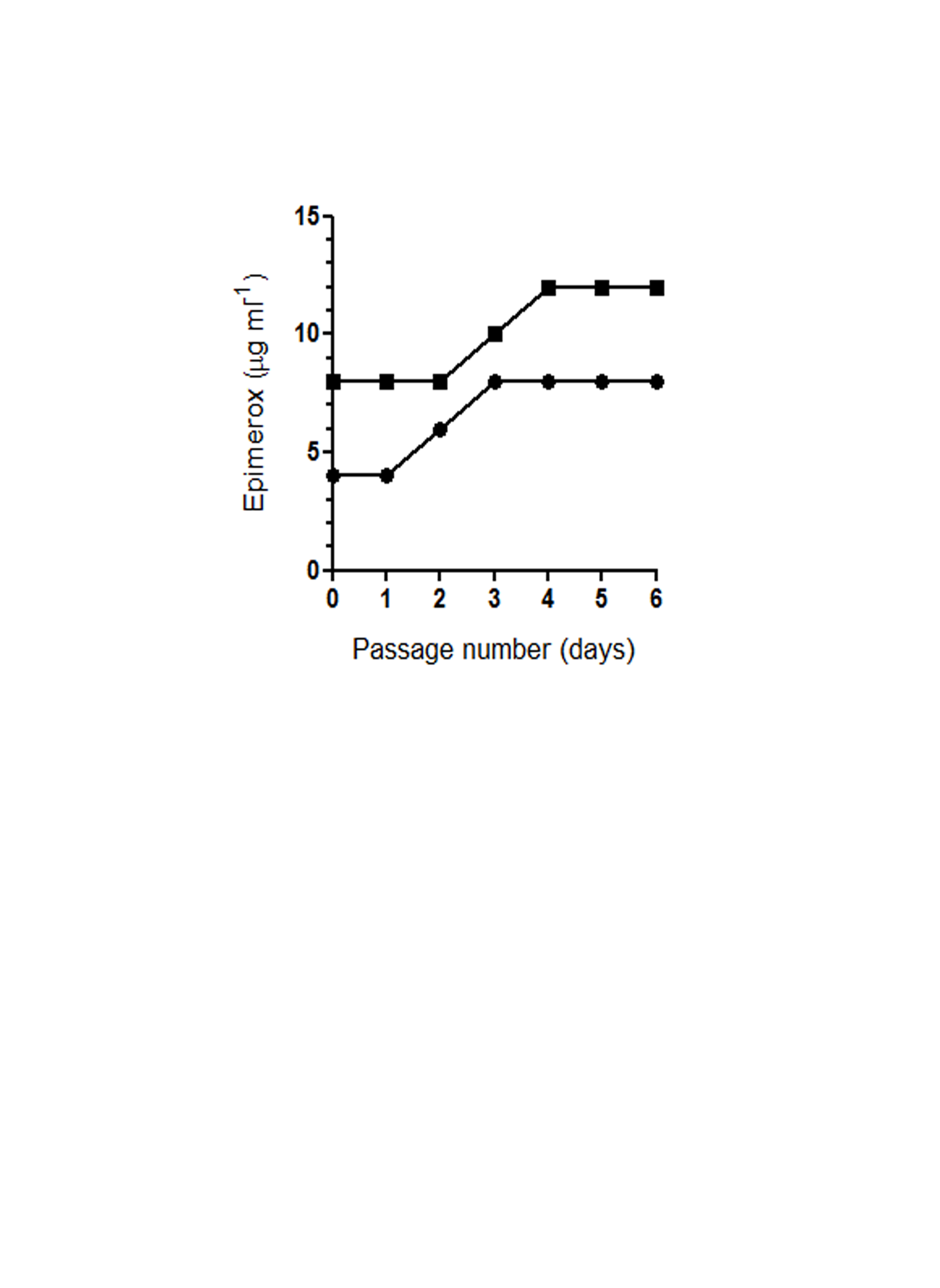

Supplement: Figure S9 — Epimerox serial passage experiments. The highest concentration of epimerox (in µg/ml) yielding growth is shown for each day of passage. No further increases were observed after six days (up to 21 days). Circles, B. anthracis Sterne; Squares, S. aureus RN4220. (TIF) [file pone.0060754.s009.tif]

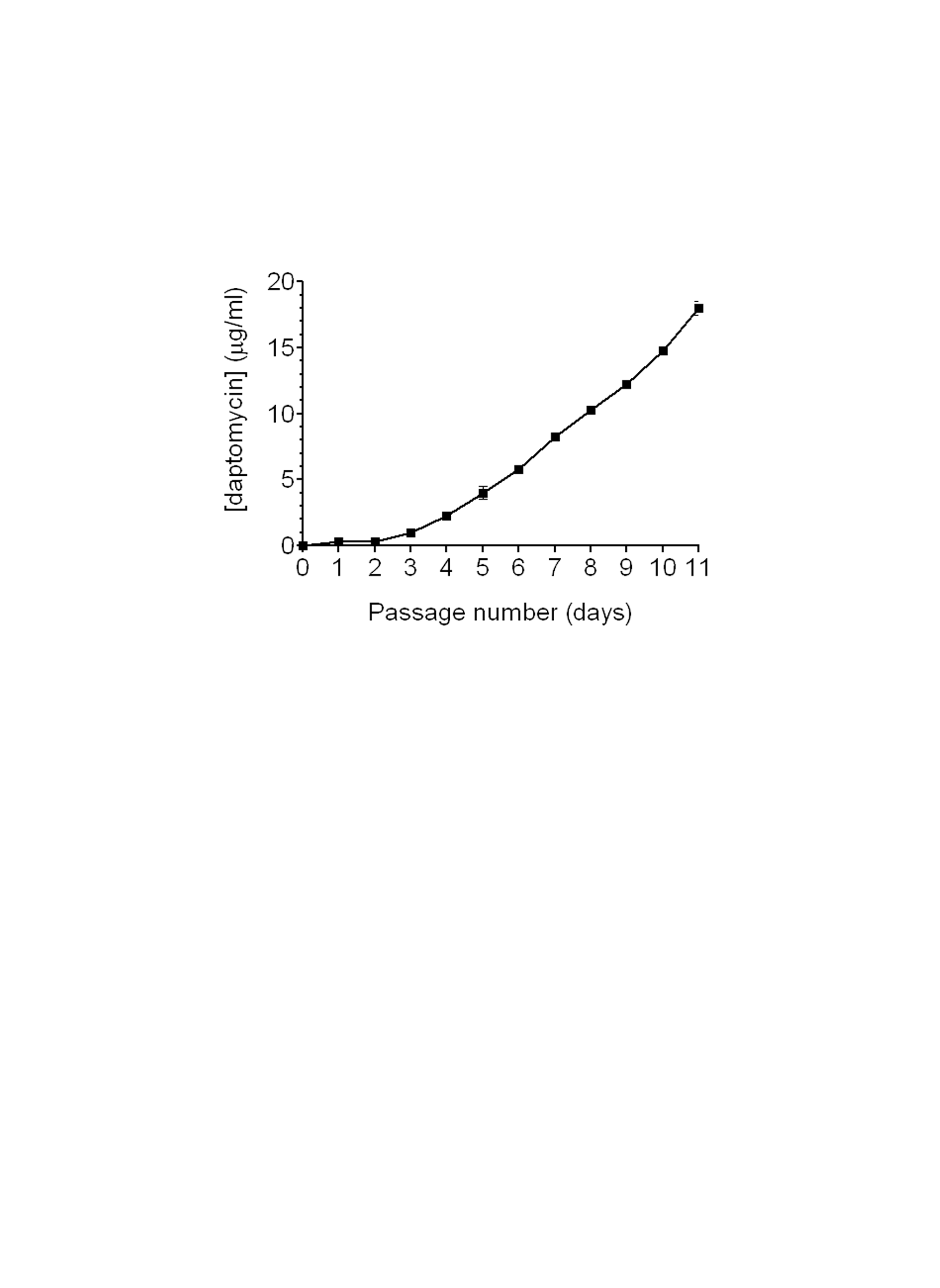

Supplement: Figure S10 — Daptomycin serial passage experiment. The highest concentration of daptomycin (in µg/ml) yielding growth of S. aureus strain RN4220 is shown for each day of passage. (TIF) [file pone.0060754.s010.tif]
