# Supplementary material for: Use of a Bacteriophage Lysin to Identify a Novel Target for Antimicrobial Development
Source: PLoS One. 2013 Apr 10;8(4):e60754. doi: 10.1371/journal.pone.0060754 (PMC3622686; doi:10.1371/journal.pone.0060754)
Supplement: File S1 — (DOCX) [file pone.0060754.s011.docx]

**Table S1.** Sugar composition of *B. anthracis* ΔSterne cell wall polysaccharide

| **Strain** | **Sugar composition (%)^A^** | | | | | |
| --- | --- | --- | --- | --- | --- | --- |
|  | **Man** | **Fuc** | **Glc** | **Gal** | **ManNAc** | **GlcNAc** |
| *B. anthracis* ΔSterne | ND | ND | ND | 49.9 | 16.5 | 33.6 |

^A^Values are expressed as mole percent of total carbohydrate. The sample was 99% carbohydrate. ND, not detected (i.e., <0.5%). Abbreviations are as follows: Man, mannose; Fuc, fucose; Glc, glucose; Gal, galactose; ManNAc, N-acetyl mannosamine; GlcNAc, N-acetyl glucosamine. Analysis was performed by combined gas chromatography/mass spectrometry (GC/MS) of the per-O-trimethylsilyl derivatives of the monosaccharide methyl glycosidase produced from the samples by acidic methanolysis.

**Table S2.** Glycosyl linkage analysis of *B. anthracis* ΔSterne cell wall polysaccharide.

| **Glycosyl residue** | **% of Material^A^** |
| --- | --- |
| Terminally linked galactopyranosyl residue (T-Gal) | 78.4 |
| 3-linked galactopyranosyl residue (3-Gal) | ND |
| 6-linked galactopyranosyl residue (6-Gal) | 1.3 |
| Terminally linked N-acetyl glucosamine residue (T-GlcNAc) | ND |
| 4-linked N-acetyl glucosamine residue (4-GlcNAc) | 4.0 |
| 4-linked N-acetyl mannosamine residue (4-ManNAc) | 2.7 |
| 6-linked N-acetyl glucosamine residue (6-GlcNAc) | ND |
| 3,4-linked N-acetyl mannosamine residue (3,4-ManNAc) | ND |
| 3,4-linked N-acetyl glucosamine residue (3,4-GlcNAc) | 10.3 |
| 4,6-linked N-acetyl glucosamine residue (4,6-GlcNAc) | 2.4 |
| 4,6-linked N-acetyl mannosamine residue (4,6-ManNAc) | ND |
| 3,4,6-linked N-acetyl mannosamine residue (3,4,6-ManNAc) | 0.9 |

^A^For glycosyl linkage analysis, the sample was permethylated, depolymerized, reduced, and acetylated; the resultant partially methylated alditol acetates (PMAAs) were analyzed by GC-MS. ND, not detected.

**Table S3.** The position and size of *sps* loci in strains from this study.

| **Organism** | **Accession**  **number** | ***sps* size (bp)** | ***sps* start^A^** | ***sps* end** |
| --- | --- | --- | --- | --- |
| *B. anthracis* Ames | AE016879 | 16,659 | 4,995,104 | 5,011,763 |
| *B. cereus* E33L | CP000001 | 16,115 | 5,056,954 | 5,073,069 |
| *B. cereus* 10987 | AE017194 | 19,866 | 4,970,969 | 4,990,835 |
| *B. cereus* 14579 | AE016877 | 16,217 | 5,174,133 | 5,190,530 |
| *B. thuringiensis* 97-27 | AE017355 | 14,713 | 5,001,698 | 5,016,411 |
| *B. thuringiensis* Al Hakam | CP000485 | 16,021 | 5,024,784 | 5,040,805 |

^A^Genomic positions (according to the indicated GenBank sequences) of the 5’ end of the first *sps* gene and the 3’ end of the last *sps* gene are reported. The *sps* locus consists of all loci between *lytR* and *mre*. The first *sps* gene for each strain is: *BA5508*, Ames; *BCZK4963*, E33L; *BCE_5384*, ATCC 10987; *BC5266*, ATCC 14579; *BT9727_4948*, 97-27, and *BALH_4769*, Al Hakam. The last *sps* gene for each strain is: *BA5519*, Ames; *BCZK4979*, E33L; *BCE_5403*, ATCC 10987; *BC5280*, ATCC 14579; *BT9727_4961*, 97-27, and *BALH_4784*, Al Hakam.

**Table S4.** Sequence comparisons of *sps* loci (and flanking regions).

| **Strain** | **% identity to *B. anthracis* Ames loci** | | | **PlyG sensitivity** |
| --- | --- | --- | --- | --- |
|  | **10 kb left^A^** | ***sps* locus** | **10 kb right^B^** |  |
| *B. cereus* E33L | 95 | 61 | 97 | + |
| *B. cereus* 10987 | 84 | 7 | 90 | - |
| *B. cereus* 14579 | 90 | 7 | 91 | - |
| *B. thuringiensis* 97-27 | 96 | 7 | 97 | - |
| *B. thuringiensis* Al Hakam | 97^C^ | 7 | 98 | - |

^A^The left end regions of homology are defined according to positions in GenBank sequences as follows: 4,981,947-4,991,947 in Ames; 5,043,808-5,053,808 in E33L; 4,956,643-4,966,643 in ATCC 10987; 5,160,984-5,170,984 in ATCC 14579; 4,988,569-4,998,569 in 97-27; and 5,006,504-5,016,504 in Al Hakam. The starting point adjacent to *sps* was chosen, in each case, as the locus immediately downstream of *galE1*.

^B^In right end regions of homology are defined as follows: 5,014,709-5,024,709 in Ames; 5,084,089-5,094,089 in E33L; 4,992,880-5,002,880 in ATCC 10987; 5,194,047-5,204,047 in ATCC 14579; 5,018,985-5,028,985 in 97-27; 5,043,242-5,053,242 in Al Hakam. The starting point adjacent to *sps* was chosen, in each case, as the 3’ end of *spoIIQ*.

^C^The left-end region of homology between Ames and Al Hakam only extends 5 kb. The value here denotes the % identity over this 5 kb region.

**Table S5.** The G+C content of *sps* loci (and it flanking regions).

| **Strain** | **% G+C** | | | |
| --- | --- | --- | --- | --- |
|  | **Total genome^A^** | **10 kb left^B^** | ***sps* locus** | **10 kb right^C^** |
| *B. anthracis* Ames | 35.4 | 37.47 | 31.95 | 37.84 |
| *B. cereus* E33L | 35.4 | 37.51 | 33.03 | 37.96 |
| *B. cereus* 10987 | 35.6 | 38.12 | 32.18 | 38.33 |
| *B. cereus* 14579 | 35.3 | 37.28 | 31.93 | 37.39 |
| *B. thuringiensis* 97-27 | 35.4 | 37.34 | 31.85 | 37.78 |
| *B. thuringiensis* Al Hakam | 35.4 | 38.45 | 32.30 | 37.89 |

^A^The total chromosomal G+C content of each strain listed here was taken from the website <http://insilico.ehu.es/oligoweb/index2.php?m=all>.

^B^The chromosomal positions of left flanking regions were identical to that listed in Supplementary Table 4. The value for the left end region of Al Hakam represents only 5 kb of flanking sequence.

^C^The chromosomal positions of right flanking regions were identical to that listed in Supplementary Table 4.

**Table S6.** Effect of 2-epimerase expression on the MIC of epimerox^A^.

| **IPTG (mM)** | ***BA5509 BA5433* mutant log_2_(s.d.)^B^** | ***p-*value** | **Epimerox MIC (μg/ml)** | **Sterne log_2_(s.d.)** | ***p-***  **value** | **Epimerox MIC (μg/ml)** |
| --- | --- | --- | --- | --- | --- | --- |
| 0 | 0 |  | 4 | 0 |  | 4 |
| 0.01 | -0.309 (0.43) | 1 | 4 | -0.65 (0.11) | 1 | 4 |
| 0.05 | 0.296 (0.43) | 0.5856 | 4 | 0.334 (0.51) | 0.4919 | 4 |
| 0.1 | 1.755 (0.69) | 0.0038 | 4 | 0.763 (0.16) | 0.0403 | 4 |
| 0.25 | 1.42 (0.18) | 0.0123 | 4 | -0.578 (0.22) | 0.2707 | 4 |
| 0.5 | 1.71 (0.32) | 0.0045 | 8 | 0.139 (0.26) | 0.9365 | 4 |
| 1 | 2.78 (0.43) | 0.0002 | 8 | 0.203 (0.14) | 0.8153 | 4 |
| 5 | 2.76 (0.37) | 0.0002 | 8 | -0.151 (0.08) | 0.0996 | 4 |
| 10 | 3.09 (0.07) | <.0001 | 8 | 0.204 (0.09) | 0.8153 | 4 |
| 25 | 3.12 (0.43) | <.0001 | 8 | 0.20 (0.29) | 0.8054 | 4 |

^A^Two sets each of the 2-epimerase double mutant (i.e., the *BA5509 BA5433* encoding the IPTG-inducible P^SPAC^-*BA5509* fusion) and the parental Sterne strain of *B. anthracis* (transformed with pNFd13 and pASD4 lacking any inserts), were grown in the presence of a range of indicated IPTG concentrations. One set was used to determine the MIC of epimerox according to the standard broth microdilution method[^1^](#_ENREF_1). The second set was grown for 5 hours prior to the extraction of RNA and processing for qRT-PCR analysis in the manner described[^2^](#_ENREF_2).

^B^Log_2_ fold changes in expression ratios are shown for each mutant relative to the control (*rpoB)* and were derived from duplicate wells in two independent experiments. Values are expressed as mean expression ratios with standard deviations (s.d.). *t-* tests were run to compare the means of IPTG induced expression as compared to control (0 mM) using the Dunnett’s method to control for multiple comparisons[^3^](#_ENREF_3). *p*-values <0.05 are considered significant.

**Table S7.** Primers used in this study.

| **Gene (study)** | **Upstream (5’-3’)** | **Downstream (5’-3’)** |
| --- | --- | --- |
| *BA5509* (RT-PCR) | Taatggcggaccttcatttc | caagaaccggtacaccaagtga |
| *BA5510* (RT-PCR) | Gttggaattgtaggtttaaatggttctg | ggaacagtggatattaaaggttcagc |
| *BA5511* (RT-PCR) | Ccagtacatggcgttccttactt | Agagctccgcgatatacttctac |
| *BA5509* (mutagenesis)^A^ | **ggggacaagtttgtacaaaaaagcaggct**- catgtataataatacagtaacaatactaccaga | **ggggaccactttgtacaagaaagctgggt**-  gaaggtccgccattacgcctg |
| *BA5433*  (mutagenesis)^B^ | gta**ggtacc**ggcacctcttgtattagagttg | gta**ggtacc**caacacgaggtttagaaggtttg |
| *BA5509* (qRT-PCR) | cgtactagagaaacttggaaataatcgtctt | gcacggaacatattacgcattgg |
| *rpoB* (qRT-PCR) | agctgaaacattagtagatccagaaactg | aatgcgatcaagtgtacgacgat |

^A^Bolded sequences represent attB1 and attB2 sites.

^B^Bolded sequences represent *Kpn*I sites.

**References**

1. Wiegand, I., Hilpert, K. & Hancock, R.E. Agar and broth dilution methods to determine the minimal inhibitory concentration (MIC) of antimicrobial substances. *Nat Protoc* **3**, 163-175 (2008).

2. Ryan, P.A., Kirk, B.W., Euler, C.W., Schuch, R. & Fischetti, V.A. Novel algorithms reveal streptococcal transcriptomes and clues about undefined genes. *PLoS Comput Biol* **3**, e132 (2007).

3. Yuan, J.S., Reed, A., Chen, F. & Stewart, C.N., Jr. Statistical analysis of real-time PCR data. *BMC Bioinformatics* **7**, 85 (2006).
